# General synthesis of 2D rare-earth oxide single crystals with tailorable facets

**DOI:** 10.1093/nsr/nwab153

**Published:** 2021-08-23

**Authors:** Linyang Li, Fangyun Lu, Wenqi Xiong, Yu Ding, Yangyi Lu, Yao Xiao, Xin Tong, Yao Wang, Shuangfeng Jia, Jianbo Wang, Rafael G Mendes, Mark H Rümmeli, Shengjun Yuan, Mengqi Zeng, Lei Fu

**Affiliations:** College of Chemistry and Molecular Sciences, Wuhan University, Wuhan 430072, China; College of Chemistry and Molecular Sciences, Wuhan University, Wuhan 430072, China; Key Laboratory of Artificial Micro- and Nano-Structures of Ministry of Education and School of Physics and Technology, Wuhan University, Wuhan 430072, China; College of Chemistry and Molecular Sciences, Wuhan University, Wuhan 430072, China; College of Chemistry and Molecular Sciences, Wuhan University, Wuhan 430072, China; The Institute for Advanced Studies, Wuhan University, Wuhan 430072, China; College of Chemistry and Molecular Sciences, Wuhan University, Wuhan 430072, China; College of Chemistry and Molecular Sciences, Wuhan University, Wuhan 430072, China; Key Laboratory of Artificial Micro- and Nano-Structures of Ministry of Education and School of Physics and Technology, Wuhan University, Wuhan 430072, China; Key Laboratory of Artificial Micro- and Nano-Structures of Ministry of Education and School of Physics and Technology, Wuhan University, Wuhan 430072, China; College of Physics, Optoelectronics and Energy, and Collaborative Innovation Center of Suzhou Nano Science and Technology, Soochow University, Suzhou 215006, China; Institute for Complex Materials, IFW Dresden, Dresden 01069, Germany; College of Physics, Optoelectronics and Energy, and Collaborative Innovation Center of Suzhou Nano Science and Technology, Soochow University, Suzhou 215006, China; Institute for Complex Materials, IFW Dresden, Dresden 01069, Germany; Centre of Polymer and Carbon Materials, Polish Academy of Sciences, Zabrze 41-819, Poland; Institute of Environmental Technology, VSB-Technical University of Ostrava, Ostrava 708 33, Czech Republic; Key Laboratory of Artificial Micro- and Nano-Structures of Ministry of Education and School of Physics and Technology, Wuhan University, Wuhan 430072, China; College of Chemistry and Molecular Sciences, Wuhan University, Wuhan 430072, China; College of Chemistry and Molecular Sciences, Wuhan University, Wuhan 430072, China; The Institute for Advanced Studies, Wuhan University, Wuhan 430072, China

**Keywords:** 2D materials, cerium, crystal engineering, rare earth, CVD

## Abstract

Two-dimensional (2D) rare-earth oxides (REOs) are a large family of materials with various intriguing applications and precise facet control is essential for investigating new properties in the 2D limit. However, a bottleneck remains with regard to obtaining their 2D single crystals with specific facets because of the intrinsic non-layered structure and disparate thermodynamic stability of different facets. Herein, for the first time, we achieve the synthesis of a wide variety of high-quality 2D REO single crystals with tailorable facets via designing a hard-soft-acid-base couple for controlling the 2D nucleation of the predetermined facets and adjusting the growth mode and direction of crystals. Also, the facet-related magnetic properties of 2D REO single crystals were revealed. Our approach provides a foundation for further exploring other facet-dependent properties and various applications of 2D REO, as well as inspiration for the precise growth of other non-layered 2D materials.

## Teaser

The synthesis of a wide variety of high-quality 2D rare-earth oxide single crystals with tailorable facets is achieved via designing a hard-soft-acid-base couple for controlling the 2D nucleation and growth of the predetermined facets.

## INTRODUCTION

Rare-earth (RE) elements possess intriguing chemical and physical properties because of their unique electron structures [1–3]. The shielded nature of *4f* electrons in RE atoms leads to highly coherent *4f*-*4f* optical and spin transitions [4,5], which makes rare-earth composites excellent candidates in the fields of luminescent [6], magnetic [7], electronic [8,9] and catalytic activities [10,11]. Two-dimensional (2D) rare-earth oxides (REOs), incorporating the unique behaviors of RE elements [12], are drawing immense interest for various applications such as optics [13], magnetism [14,15], high-efficiency catalysts [16,17], transistors [18,19] and biomedicine [15]. Owing to the enhanced surface-area-to-volume ratio and quantum confinement [12,13,17], 2D REOs are highly promising with regard to showing diverse properties, some of which do not exist in bulk materials. Meanwhile, the delicate manipulation of exposed facets of 2D materials greatly provides an effective strategy for designing novel metal oxide micro-/nanostructures with a high degree of freedom, and enlarging the facet-related properties of the materials, thus enriching fundamental and practical research [20,21]. It has been reported that the crystallographic orientation and surface energy (*γ*) of REOs will influence their optical, electronic, hydrophobic, magnetic and catalytic properties [5,22–24]. Thus, it has become a vital issue to controllably synthesize various ultrathin 2D REO single crystals with predetermined facets and explore their unique properties and potential applications.

However, delicately manipulating the thermodynamic stability of the 2D REO exposed facets to form specific architectures is still a challenge. For one thing, the inherent strong ionic bonding in the non-layered cubic structures [4,12] of the REO compounds hinders the exfoliation process, and even worse, it easily induces an isotropic growth along three dimensions, thus greatly impeding the 2D anisotropic growth. For another thing, the accessible 2D crystals are dominated by the thermodynamically stable facets with lower *γ*, which impedes the generation of 2D crystals exposing other high-active facets [25,26]. Strategies were established to regulate the stability of corresponding surfaces by using organic surfactants [27–29], mineralizers [30] or foreign chemicals [31] in the solution system, which can realize the controllable design of the nanocrystal facet [26,32]. Unfortunately, the introduced organic residues may affect the availability of high-quality crystals. As a consequence, the development of a universal and facile method to effectively synthesize a series of high-quality 2D REO single crystals with tailorable facets is important but challenging.

Herein, for the first time, we present a general method for synthesizing various high-quality 2D REO single crystals covering light REO to heavy REO, tailoring their facets and exploring the facet-related properties that were impossible in the chemical vapor deposition (CVD) system before. 2D REO single crystals exposing different facets were produced from controllable dissolution and precipitation in natural solvent liquid gold (Au) directed by a facet-controlling assistor (FCA) according to the designing principle of a hard-soft-acid-base (HSAB) couple. Density functional theory (DFT) calculations verified that the *γ* of different REO facets would reverse with the increase of FCA and the exposed facet would change from (111) to (100). Our versatile work brings forth new insights for realizing the anisotropic growth of non-layered 2D REO materials and enriches the 2D material family. Notably, the establishment of a general synthesis method and the high facet maneuverability of 2D REO single crystals open up opportunities for studying a wide range of properties and potential applications.

## RESULTS AND DISCUSSION

### Universal synthesis of 2D REO single crystals with controllable facets

**Figure 1.**
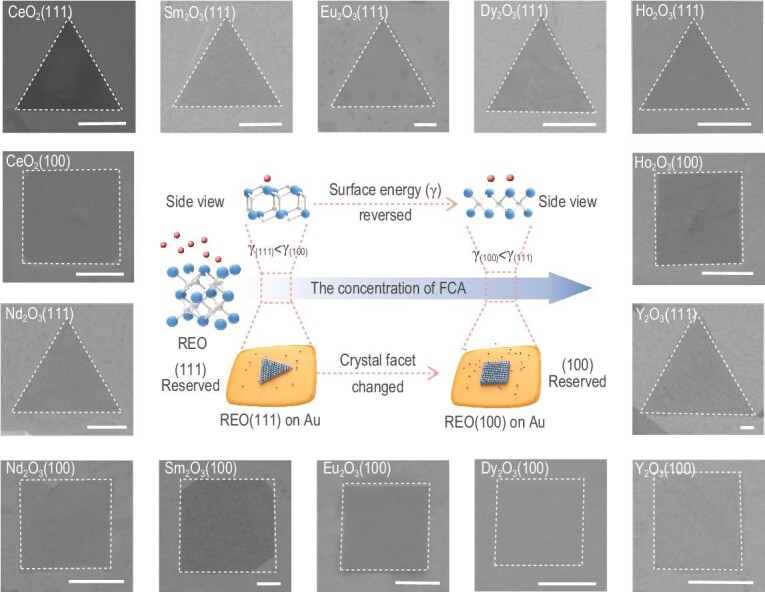
Schematic illustration of the universality of the facet-controllable synthesis strategy assisted by the FCA. The schematic in the middle shows the growth process of 2D REO single crystals with tailorable facets (blue atoms, RE atoms; white atoms, O atoms; red atoms, X ions (base)). The SEM images around the schematic show the as-obtained 2D REO single crystals exposing different facets. Scale bar: 1 μm.

2D REO single crystals exposing different facets were successfully produced which can be attributed to the following aspects: (i) REOs have good solubility and non-reactivity in liquid Au, which is conducive to their saturated precipitation and nucleation on the Au surface during cooling and also can avoid residual impurities compared with the common organic solvent system; (ii) during the growth process, the strong interaction of the introduced FCA with the REO surface greatly manipulates the growth mode and direction of the non-layered REO (see Methods). Based on the HSAB theory [33], RE ions are hard acid and prefer to have affinities toward the base. Here, NH_4_X (X^−^ = Cl^−^, Br^−^, I^−^) is employed as the FCA, and halide ions (X^−^) belonging to the base act as the active assistor. The introduction of an FCA not only suppresses the isotropic growth along the three-dimensional (3D) direction and impedes the thickening of 2D REOs, but also leads to the change in the relative γ of each facet with the increasing concentration of FCA and eventually determines the final exposing facet (Fig. 1). The strategy can be extended to the facet-controllable synthesis of a series of 2D REO single crystals, including light REO (CeO_2_, Nd_2_O_3_), middle REO (Sm_2_O_3_, Eu_2_O_3_) and heavy REO (Dy_2_O_3_, Ho_2_O_3_, Y_2_O_3_), respectively (Fig. 1). We use Raman spectroscopy to verify the crystals we obtained. Scanning electron microscopy (SEM) and energy-dispersive X-ray spectroscopy (EDS) mappings of these REO crystals with different facets are also presented to confirm their uniformity (Figs S1–S7). For convenience, all we show here are the results about the facet-controllable effect of FCA_Cl_. In addition, by controlling the reaction time and temperature, we can also regulate the lateral size of the single crystals (Figs S8 and S9). The universal facet-controllable growth of 2D CeO_2_ single crystals by introducing FCA_Br_ and FCA_I_ are shown in Figs S10 and S11.

### High-quality 2D CeO_2_(111) and CeO_2_(100) single crystals

**Figure 2.**
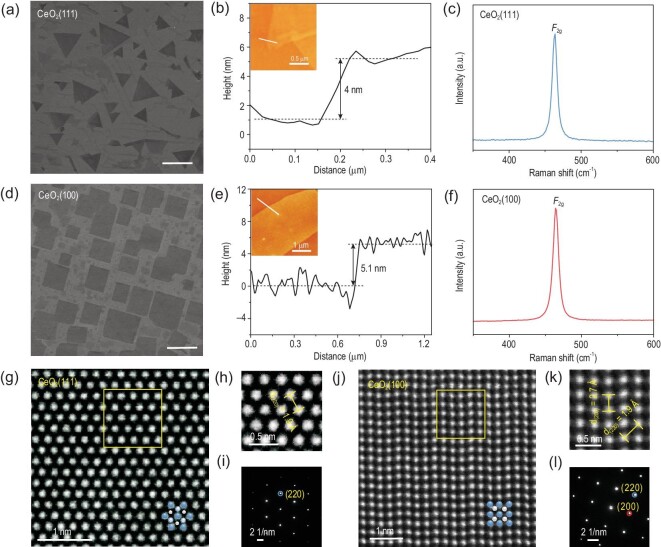
Morphological and atomic morphology of the as-synthesized 2D CeO_2_ single crystals. (a and d) SEM images of trigonal and square 2D CeO_2_ single crystals; scale bar: 5 μm. (b and e) AFM images of the trigonal CeO_2_ single crystal with a thickness of 4 nm and the square CeO_2_ single crystal with a thickness of 5.1 nm. (c and f) Typical Raman spectra of trigonal and square 2D CeO_2_ single crystals. (g and j) HAADF-STEM images of 2D CeO_2_(111) and CeO_2_(100) single crystals, respectively (blue atoms: Ce; white atoms: O). (h and k) The magnified STEM images from the yellow box regions in (g and j), respectively. (i and l) SAED patterns of 2D CeO_2_(111) and CeO_2_(100) single crystals, respectively.

We employed the as-synthesized 2D CeO_2_ single crystals with different exposed facets as a typical example to conduct further characterization for benchmarking our approach. As mentioned above, the facet of CeO_2_ was sensitive to the concentration of FCA_Cl_. Increasing the concentration of FCA_Cl_ will lead to the facet transition from CeO_2_(111) to CeO_2_(100), with the shape changing from triangle to square (Fig. 2a and d). The atomic force microscopy (AFM) image in Fig. 2b shows an individual triangular CeO_2_(111) flake with a thickness of 4 nm. As for square CeO_2_(100), the thickness was 5.1 nm (Fig. 2e). The Raman spectrum of a triangular flake showed a single peak at ∼463 cm^−1^ attributing to the *F*_2g_ mode of cubic phase CeO_2_ (Fig. 2c), and the symmetry and strength of *F*_2g_ confirmed the high crystallinity of the 2D CeO_2_(111) single crystal. A similar result was also observed in the Raman spectrum of the square 2D CeO_2_(100) single crystal (Fig. 2f) [34]. Figure S12 shows X-ray diffraction (XRD) spectra for 2D CeO_2_ single crystals respectively, suggesting the successful synthesis of pure 2D CeO_2_ single crystals exposing specific pure facets. The exhibition of Au(111) in both cases verified that the facet-controllable process is not derived from the epitaxy growth of Au substrate. Based on the low-loss electron energy loss spectra (EELS) (Fig. S13), the bandgap of the 2D CeO_2_(111) and CeO_2_(100) were estimated to be 5.0 eV and 4.98 eV, which showed a distinct blue shift compared with bulk CeO_2_ (3.2 eV) [13]. The increased bandgap for the 2D CeO_2_ single crystal indicated the existence of a strong quantum size effect in the 2D limit. In addition, the X-ray photoelectron spectroscopy (XPS) spectra and analyses (Fig. S14a and b) demonstrated that both 2D CeO_2_(111) and CeO_2_(100) single crystals are reasonably stoichiometric [35,36]. The XPS spectra of the 2D CeO_2_ single crystals exposing different facets by employing NH_4_Br and NH_4_I as FCA were also collected in Figs S15 and S16.

To probe the atomic structural differences of CVD-grown 2D CeO_2_(111) and CeO_2_(100) single crystals, high-angle annular dark-field scanning transmission electron microscopy (HAADF-STEM) images were utilized to reveal the atomic arrangement of the crystals (Fig. 2g and j). Also, bright-field STEM (BF-STEM) images were exhibited in Fig. S17. In Fig. 2h, the lattice distance of the 2D CeO_2_(111) crystal was measured to be ∼1.9 Å, which was in good agreement with the standard PDF card (JPCDS card no. 34-0394). Figure S17b shows the intensity profile derived from the region marked by a blue line in Fig. S17a; the distance between each neighboring peak was measured to be ∼2.2 Å, which corresponded to the lattice distance of (220) facet. The EDS in Fig. S17c further implied the existence of Ce and O. The corresponding selected area electron diffraction (SAED) patterns showed an array of spots with a 6-fold and 4-fold rotational symmetry, respectively (Fig. 2i and l). The lattice distances of 2D CeO_2_(100) crystal were measured as ∼2.7 Å and ∼1.9 Å (Fig. 2k). We also further derived the lattice distance by analyzing the peak position distance (Fig. S17e and f) in the intensity profile corresponding to the region marked by the red and blue lines in Fig. S17d. The measured values agreed well with those obtained from the SAED pattern. Besides, the SAED patterns recorded from different regions of the corresponding 2D CeO_2_(111) and CeO_2_(100) crystals verified the good single crystallinity owing to the same spot spacing and coincident orientation of the patterns (Fig. S18). All of these characterizations demonstrated the atomic-thickness nature and excellent crystallinity of the 2D CeO_2_ single crystals grown via the CVD process assisted by FCA.

### Mechanism of the strategy

**Figure 3.**
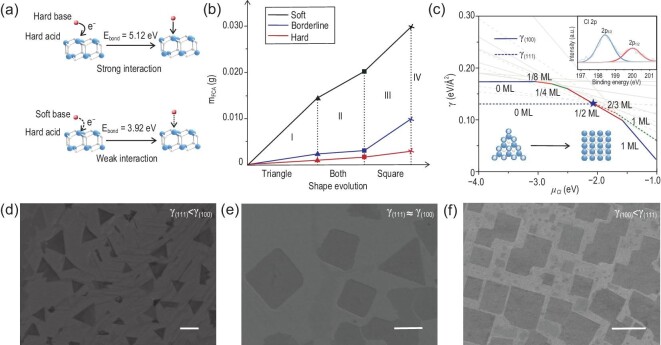
Mechanism of the facet-controllable strategy assisted by the FCA. (a) The interaction between hard acid (RE ions) and different bases (X ions). (b) Shape evolution of CeO_2_ single crystals by introducing different bases (red line: hard base; blue line: borderline base; black line: soft base; area I: triangle; area II: triangle and square; area III: square; area IV: no crystals). (c) Surface energy *γ* of CeO_2_(111) and CeO_2_(100) for different surface coverages of Cl atoms as a function of chloride chemical potential *μ_Cl_*. Each line represents one Cl coverage situation. The inset shows the Cl 2p XPS of 2D CeO_2_ single crystals exposing (100) facet obtained by the assistance of FCA_Cl_. Details are given in the Supplementary Data. Colored regions of the plot represent the minimum *γ* on each surface. Minimum surface energies on CeO_2_(100) and CeO_2_(111) are shown with solid and dashed lines, respectively. When *μ_Cl_* reaches the value of –2.05 eV (marked by a blue star), the surface energy is reversed. (d–f) Evolution of the exposed facets of 2D CeO_2_ with the increasing concentration of FCA_Cl_ (scale bars: 2 μm; (d) *γ*_(111)_ < *γ*_(100)_; (e) *γ*_(100)_ ≈ *γ*_(111)_; (f) *γ*_(100)_ < *γ*_(111)_).

FCA is a critical factor in this facet-controllable process. NH_4_X (X^−^ = Cl^−^, Br^−^, I^−^) was employed here as the FCA, and can easily supply active assistor X ions (base) to control the growth of single crystals in the corresponding reaction zone (500–600^o^C) because of the low melting point (338–551^o^C). Simultaneously, the control experiment of only introducing NH_3_, which is one of the main thermally decomposed products of NH_4_Cl, verified that NH_3_ cannot act as FCA to effectively realize the control of the exposed crystal facet (Fig. S19). RE ions have empty orbitals and prefer to accept electrons. Halide ions could form a coordinate bond by donating electrons to the empty orbitals of the metal (X^−^ (*np*)→Ln^4+^ (*5d, 4f*)). According to the HSAB theory [33], RE ions are classified as hard acid, and active ions (Cl^−^, Br^−^, I^−^) are hard base, borderline base and soft base, respectively. Due to the interaction between FCA and REO surface, the absorption of FCA on that particular facet will hinder the further precipitation of RE and O atoms in 3D dimensions, thereby promoting and obtaining 2D REO. The early nucleation of 2D CeO_2_(111) and CeO_2_(100) single crystals are shown in Fig. S20. They tend to arrange in a directional way due to the use of liquid metal substrate, which tends to become single crystal after curing, which will affect the arrangement of crystals in subsequent growth stages. The thickness was characterized to be 2–3 nm which means that we have successfully controlled the 2D nucleation of the predetermined facets of the non-layered REO (Fig. S21). Then, as the concentration of FCA increased, the chemical potential of X ions (*μ_x_*) increased, which led to the reversed *γ* ranking of each REO facet and the acquisition of 2D REOs exposing different facets. In addition, the interaction between the RE ions and X ions decreased because of the lower bond energy in sequence (Fig. 3a) [37]. Thus, the weaker the alkalinity of the active X ions, the higher corresponding FCA concentration is required to realize the 2D growth and facet-controllability of the REO (Table S1). Figure 3b shows the quantitative results and the corresponding shape evolution of CeO_2_ single crystals by introducing different bases. The shape changed from triangle to square from area I to III, but in area IV no crystals were obtained because we could not get a well-spread liquid Au substrate (Fig. 3d–f). The shape evolution process of CeO_2_ single crystals by introducing FCA_Br_ and FCA_I_ are shown in Figs S22 and S23.

It is reported that facets with lower specific *γ* are favored by thermodynamics and will be exposed on the surface of the crystal [25,26]. To confirm the mechanism further, DFT calculations were performed (Figs S24–S26). According to previous literature [38,39], *γ* can be evaluated by

(1) \begin{equation*} \gamma = \left( {{E_{slab}} - \frac{{{N_{slab}}}}{{{N_{bulk}}}}{E_{bulk}}} \right) \Big/2A,\ \end{equation*}

where *E_slab_* is the total energy of the slab, *E_bulk_* is the energy of the bulk unit cell containing the same number of atoms as in the slab, *N_slab_* is the number of atoms in the slab, *N_bulk_* is the number of atoms in the bulk, *A* is the surface area, and the factor of 2 in the denominator accounts for the two sides of the slab.

In this work, the one-sided relaxation and the influence of Cl should be considered, thus *μ_Cl_* is incorporated into the surface-energy calculation. *γ* can be donated by

(2) \begin{eqnarray*} \gamma &=& \left(\! {{E_{slab + Cl,\ relax }}\! -\! \frac{{{N_{slab}}}}{{{N_{bulk}}}}{E_{bulk}}\! -\! {N_{Cl}}{\mu _{Cl}}}\! \right)\Big/A\nonumber\\ && - {\gamma _{frozen}},\end{eqnarray*}

(3) \begin{equation*} {\gamma _{frozen}} = \left( {{E_{slab, frozen\ }} - \frac{{{N_{slab}}}}{{{N_{bulk}}}}{E_{bulk}}} \right)\Big/2{\rm{A\ }}, \end{equation*}

where *E_slab+Cl__, relax_* is the total energy of a relaxed slab with adsorbed Cl, *N_Cl_* is the number of adsorbed Cl atoms and *γ_frozen_* is the surface energy of the surface with atom positions frozen to their bulk values.

We take the FCA_Cl_ to assist the facet-controllable growth of CeO_2_ single crystals as an example. It is shown that *γ* can be plotted as a function of *μ_Cl_* to further explore the mechanism (Fig. 3c). For *μ_Cl_* < –3.04 eV, the *γ* of bare CeO_2_(111) is lower than that of CeO_2_(100). The bare CeO_2_(111) surface is thermodynamically favored in this regime, and the shape of the as-grown crystal is a triangle. However, once FCA is introduced in the system, the *γ* of CeO_2_(100) decreases significantly. As *μ_Cl_* reaches –2.05 eV, the surface coverage of Cl is 0.5 monolayer (ML) on CeO_2_(100) and 0.66 ML on CeO_2_(111), and the *γ* of CeO_2_(100) becomes lower than that of CeO_2_(111). At this point, the shape of the as-grown CeO_2_ crystal begins to change from a triangle to a square (Fig. 3c). That is, with a low *μ_Cl_*, the calculated *γ*_(111)_ is lower than *γ*_(100)_, which means exposure of the CeO_2_(111) facet is preferred. With the increase of *μ_Cl_*, the calculated *γ* of the CeO_2_ facet changes from *γ*_(111)_ < *γ*_(100)_ to *γ*_(100)_ < *γ*_(111)_ and the corresponding crystal morphology changes from triangle to square (Fig. 3d–f). Interestingly, we also found that two types of facets can be produced simultaneously, which may be attributed to the competitive reaction caused by the almost equal *γ* of these two facets at the critical point (Fig. 3e). Meanwhile, the weak signal of Cl on 2D CeO_2_(100) single crystals has been identified by XPS in Fig. 3c (inset), which demonstrates the existence of the FCA (FCA_Cl_). In addition, the Ce 3d peak shifted to the lower energy direction (∼2 eV) after the adsorption of Cl^−^ on the crystal surface, which confirmed the electron transfer from hard base (Cl^−^) to hard acid (Ce^4+^) (Fig. S14). These DFT calculation results agree well with our experimental results, which shows that the facet-controllable synthesis of materials can be successfully achieved by our method.

### Magnetic properties of 2D CeO_2_(111) and CeO_2_(100) single crystals

**Figure 4.**
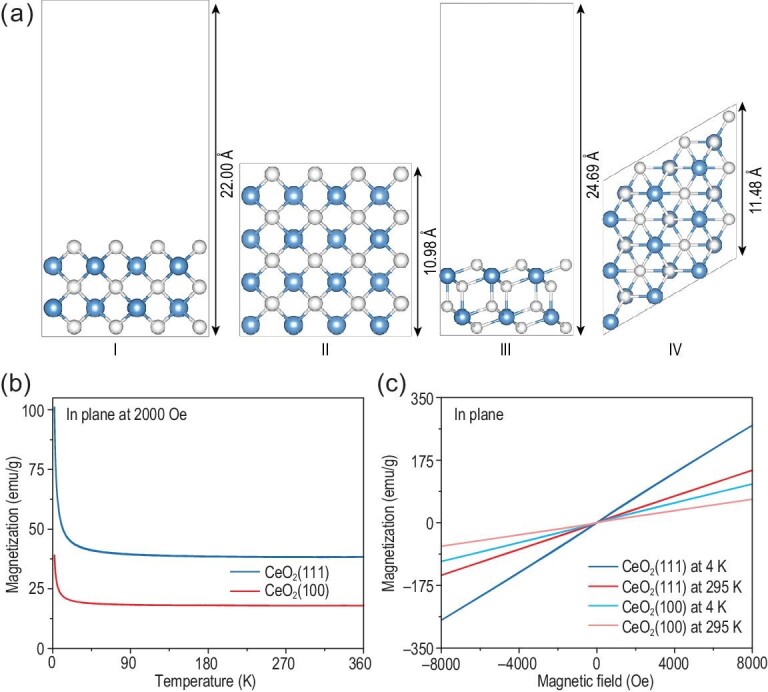
Magnetic characterization of CeO_2_ single crystals. (a) Side and top views of slab models for the two different facets of CeO_2_. (I and II) for CeO_2_(100) facet, and (III and IV) for CeO_2_(111) facet. The blue and white spheres stand for Ce and O atoms, respectively. (b) In-plane temperature-dependent magnetization of CeO_2_(111) and CeO_2_(100) single crystals at 2000 Oe. (c) In-plane magnetic hysteresis (M–H) loops of CeO_2_(111) and CeO_2_(100) single crystals at different temperatures.

To investigate the facet-dependent magnetic properties, magnetic measurements on both the 2D CeO_2_(111) and CeO_2_(100) single crystals were employed. First, a field cooling process was conducted under an external field of 2000 Oe. As shown in Fig. 4b, the temperature-dependent magnetization (M–T) indicated the obvious paramagnetic nature and the distinct different magnetization of 2D CeO_2_(111) and CeO_2_(100) single crystals, which can be attributed to the different number of Ce atoms per unit area in the terminated CeO_2_(100) and CeO_2_(111) facets [40,41]. And the estimated values were 0.066 Ce Å^–2^ and 0.079 Ce Å^–2^ for CeO_2_(100) and CeO_2_(111) facets, respectively (Fig. 4a). To quantitatively compare the magnetization, we produced statistics on the sample coverage of different crystal facets (Tables S4 and S5) and their corresponding thickness (Fig. S28). The weight of 2D CeO_2_(111) and CeO_2_(100) single crystals can be measured at ∼6.98 × 10^–7^ g and 1.34 × 10^–6^ g. Hence, the saturation magnetization of 2D CeO_2_(111) and CeO_2_(100) can be estimated to be 38.19 and 17.88 emu/g at 295 K, respectively. The magnetic hysteresis loops from 2 to 360 K presented as a straight line, indicating the paramagnetic property (Fig. 4c). Similar tendencies were shown in Fig. S27 when applying a vertical magnetic field. And the saturation moment of 2D CeO_2_(111) and CeO_2_(100) can be estimated to be ∼37.80 and 16.10 emu/g at 295 K, respectively. According to the magnetic hysteresis loops, the magnetic susceptibility decreased as the temperature increased, conforming to Curie–Weiss law, and verified the intrinsic paramagnetic nature of 2D CeO_2_ single crystals further.

## CONCLUSION

In summary, we developed a strategy to achieve a general synthesis of a series of high-quality 2D REO single crystals with tailorable facets, and also explored the intrinsic facet-dependent characteristics of 2D REO single crystals. In addition, we proposed, for the first time, that the thermodynamics of facets of 2D ultrathin materials can be manipulated by introducing FCA, which can control the 2D nucleation of the predetermined facets in the CVD process, which was previously impossible. The mechanism we demonstrated not only lays a foundation for enriching the library of 2D REOs with different facets, but also has the potential to obtain other RE composites or even other non-layered materials with every kind of facet, even high-index facets possessing enhanced chemical activities, by designing appropriate FCA. Moreover, other liquid metals like Ga, In and Sn with a lower melting point can be applied for substrates during the process of low-temperature growth of materials, which will largely reduce cost and energy consumption.

## METHODS

### Synthesis of 2D REO single crystals

The CVD growth of 2D REO single crystals was conducted in a quartz tube with a length of 1.1 m and a diameter of 25 mm, which was placed in a single hot-wall furnace (Model HTF 55322C, Lindberg/Blue M) under ambient pressure. The Au wire (3 mm) and REO powders were placed on the fresh Mo foil. The temperature of the furnace was elevated from room temperature to 1080°C (∼25°C/min) in Ar (200–300 sccm) and H_2_ (5–20 sccm) atmosphere and maintained at 1065–1080°C for 10–25 min to ensure Au spread evenly over the entire foil and to allow REO powders to dissolve into the molten Au. After that, the temperature was naturally decreased to room temperature under Ar/H_2_ flow. Either zero, or only a few grains, of NH_4_X (X^−^ = Cl^−^, Br^−^, I^−^) were put in a quartz boat and placed at the upstream of the quartz tube, ∼10 cm away from the substrate, to induce the synthesis of 2D REO (111) single crystals. For the growth of 2D REO (100) single crystals, more NH_4_X was needed. The quantitative experiment of the growth 2D CeO_2_ single crystals is shown in Table S1. Upon adsorption of X ions on REO facets, the surface energies of different REO facets were reversed to be *γ*_(100)_ < *γ*_(111)_ and, thermodynamically, the exposure of the REO (100) facet was preferred.

### Characterization

Raman spectra were conducted using a Renishaw-inVia Plus with an excitation wavelength of 532 nm. SEM images were taken by Zeiss Sigma. XPS was performed on a Thermo Scientific, ESCALAB 250Xi. The binding energies were calibrated by referencing the C 1s peak (284.8 eV). The AFM image was measured on a confocal laser microscope system (Alpha 300RS+, WITec). The XRD pattern was measured on a Rigaku Miniflex600 powder diffractometer. The TEM images in Fig. 2 were obtained with JEOL COM operated at 80 kV. The low-loss electron EELS spectra were obtained by FEI Tecnai operated at 80 kV. The TEM images in Fig. S18 were obtained by a probe-corrected high-resolution TEM system (FEI Tecnai) operated at 300 kV. All magnetic measurements were carried out in a magnetic property measurement system (MPMS–XL, Quantum Design).

## Supplementary Material

nwab153_Supplemental_File

## References

[1] Cheisson T , Schelter EJ. Rare earth elements: Mendeleev's bane, modern marvels. Science 2019; 363: 489–93. 10.1126/science.aau7628 30705185

[2] Fortner JA , Buck EC. The chemistry of the light rare-earth elements as determined by electron energy loss spectroscopy. Appl Phys Lett 1996; 68: 3817–9. 10.1063/1.116627

[3] Chen P , Han W, Zhao M et al. Recent advances in 2D rare earth materials. Adv Funct Mater 2020; 31: 2008790. 10.1002/adfm.202008790

[4] Adachi G-y , Imanaka N. The binary rare earth oxides. Chem Rev 1998; 98: 1479–514. 10.1021/cr940055h 11848940

[5] Azimi G , Dhiman R, Kwon HM et al. Hydrophobicity of rare-earth oxide ceramics. Nat Mater 2013; 12: 315–20. 10.1038/nmat3545 23333998

[6] Binnemans K , Moors D. Narrow band photoluminescence of europium-doped liquid crystals. J Mater Chem 2002; 12: 3374–6. 10.1039/b206810a

[7] Guo F-S , Day BM, Chen Y-C et al. Magnetic hysteresis up to 80 kelvin in a dysprosium metallocene single-molecule magnet. Science 2018; 362: 1400–3. 10.1126/science.aav0652 30337456

[8] Zhang J , Hess PW, Kyprianidis A et al. Observation of a discrete time crystal. Nature 2017; 543: 217–20. 10.1038/nature21413 28277505

[9] Zhang J , Pagano G, Hess PW et al. Observation of a many-body dynamical phase transition with a 53-qubit quantum simulator. Nature 2017; 551: 601–4. 10.1038/nature24654 29189781 PMC6506159

[10] Wang Y-G , Mei D, Glezakou V-A et al. Dynamic formation of single-atom catalytic active sites on ceria-supported gold nanoparticles. Nat Commun 2015; 6: 6511. 10.1038/ncomms7511 25735407 PMC4366521

[11] Nie L , Mei D, Xiong H et al. Activation of surface lattice oxygen in single-atom Pt/CeO_2_ for low-temperature CO oxidation. Science 2017; 358: 1419–23. 10.1126/science.aao2109 29242344

[12] Xu J , Chen X, Xu Y et al. Ultrathin 2D rare-earth nanomaterials: compositions, syntheses, and applications. Adv Mater 2020; 32: e1806461. 10.1002/adma.201806461 31018020

[13] Yu T , Lim B, Xia Y. Aqueous-phase synthesis of single-crystal ceria nanosheets. Angew Chem Int Ed 2010; 49: 4484–7. 10.1002/anie.201001521 20458722

[14] Schmitt R , Spring J, Korobko R et al. Design of oxygen vacancy configuration for memristive systems. ACS Nano 2017; 11: 8881–91. 10.1021/acsnano.7b03116 28850213

[15] Jeong J , Kim N, Kim M-G et al. Generic synthetic route to monodisperse sub-10 nm lanthanide oxide nanodisks: a modified digestive ripening process. Chem Mater 2016; 28: 172–9. 10.1021/acs.chemmater.5b03616

[16] Wang D , Kang Y, Doan-Nguyen V et al. Synthesis and oxygen storage capacity of two-dimensional ceria nanocrystals. Angew Chem Int Ed 2011; 50: 4378–81. 10.1002/anie.201101043 21480452

[17] Sun Y , Liu Q, Gao S et al. Pits confined in ultrathin cerium(IV) oxide for studying catalytic centers in carbon monoxide oxidation. Nat Commun 2013; 4: 2899. 10.1038/ncomms3899 24280902

[18] Hong M , Kwo J, Kortan AR et al. Epitaxial cubic gadolinium oxide as a dielectric for gallium arsenide passivation. Science 1999; 283: 1897–900. 10.1126/science.283.5409.1897 10082459

[19] Addou R , Dahal A, Batzill M. Growth of a two-dimensional dielectric monolayer on quasi-freestanding graphene. Nat Nanotechnol 2013; 8: 41–5. 10.1038/nnano.2012.217 23263724

[20] Vile G , Colussi S, Krumeich F et al. Opposite face sensitivity of CeO_2_ in hydrogenation and oxidation catalysis. Angew Chem Int Ed 2014; 53: 12069–72. 10.1002/anie.201406637 25146728

[21] Yang C , Yu X, Heißler S et al. O_2_ activation on ceria catalysts—the importance of substrate crystallographic orientation. Angew Chem Int Ed 2017; 56: 16399–404. 10.1002/anie.201709199 29024254

[22] Bruix A , Lykhach Y, Matolinova I et al. Maximum noble-metal efficiency in catalytic materials: atomically dispersed surface platinum. Angew Chem Int Ed 2014; 53: 10525–30. 10.1002/anie.201402342 24919780

[23] Daelman N , Capdevila-Cortada M, Lopez N. Dynamic charge and oxidation state of Pt/CeO_2_ single-atom catalysts. Nat Mater 2019; 18: 1215–21. 10.1038/s41563-019-0444-y 31384029

[24] Zhou Z , Hu R, Wang L et al. Water bridge coordination on the metal-rich facets of Gd_2_O_3_ nanoplates confers high T1 relaxivity. Nanoscale 2016; 8: 17887–94. 10.1039/C6NR06444B 27722744 PMC5073006

[25] Xia Y , Xia X, Peng H-C. Shape-controlled synthesis of colloidal metal nanocrystals: thermodynamic versus kinetic products. J Am Chem Soc 2015; 137: 7947–66. 10.1021/jacs.5b04641 26020837

[26] Ghosh S , Manna L. The many ‘facets’ of halide ions in the chemistry of colloidal inorganic nanocrystals. Chem Rev 2018; 118: 7804–64. 10.1021/acs.chemrev.8b00158 30062881 PMC6107855

[27] Zhong Y , Yang Y, Ma Y et al. Controlled synthesis of ultrathin lamellar Eu_2_O_3_ nanocrystals: self-assembly of 1D nanowires to 2D nanosheets. Chem Commun 2013; 49: 10355–7. 10.1039/C3CC43673J 23872870

[28] Zhang Q , Yan B. Salt-effect-based synthesis and anomalous magnetic properties of rare-earth oxide nanosheets with sub-1nm thickness. Chem Eur J 2012; 18: 5150–4. 10.1002/chem.201103596 22431269

[29] Sun Y , Xia Y. Shape-controlled synthesis of gold and silver nanoparticles. Science 2002; 298: 2176–9. 10.1126/science.1077229 12481134

[30] Wang D , Kang Y, Ye X et al. Mineralizer-assisted shape-control of rare earth oxide nanoplates. Chem Mater 2014; 26: 6328–32. 10.1021/cm502301u

[31] Wang H , Rogach AL. Hierarchical SnO_2_ nanostructures: recent advances in design, synthesis, and applications. Chem Mater 2014; 26: 123–33. 10.1021/cm4018248

[32] Chen C , Hu R, Mai K et al. Shape evolution of highly crystalline anatase TiO_2_ nanobipyramids. Cryst Growth Des 2011; 11: 5221–6. 10.1021/cg200457g

[33] Pearson RG . Acids and bases. Science 1966; 151: 172–7. 10.1126/science.151.3707.172 17746330

[34] Maensiri S , Masingboon C, Laokul P et al. Egg white synthesis and photoluminescence of platelike clusters of CeO_2_ nanoparticles. Cryst Growth Des 2007; 7: 950–5. 10.1021/cg0608864

[35] Paparazzo E , Ingo GM, Zacchetti N. X-ray induced reduction effects at CeO_2_ surfaces: an X-ray photoelectron spectroscopy study. J Vac Sci Technol A 1991; 9:1416–20. 10.1116/1.577638

[36] Burroughs P , Hamnett A, Orchard AF et al. Satellite structure in the X-ray photoelectron spectra of some binary and mixed oxides of lanthanum and cerium. J Chem Soc, Dalton Trans 1976: 1686–98. 10.1039/dt9760001686

[37] Ji W-X , Xu W, Xiao Y et al. Does the 4f-shell contribute to bonding in tetravalent lanthanide halides? J Chem Phys 2014; 141: 244316. 10.1063/1.4904722 25554160

[38] Yang Z , Woo TK, Baudin M et al. Atomic and electronic structure of unreduced and reduced CeO_2_ surfaces: a first-principles study. J Chem Phys 2004; 120: 7741–9. 10.1063/1.1688316 15267687

[39] Chen Z , Balankura T, Fichthorn KA et al. Revisiting the polyol synthesis of silver nanostructures: role of chloride in nanocube formation. ACS Nano 2019; 13: 1849–60. 30673260 10.1021/acsnano.8b08019

[40] Ge M , Wang H, Liu E et al. On the origin of ferromagnetism in CeO_2_ nanocubes. Appl Phys Lett 2008; 93: 062505. 10.1063/1.2972118

[41] Luo T , Meng Q-Q, Gao C et al. Sub-20 nm-Fe_3_O_4_ square and circular nanoplates: synthesis and facet-dependent magnetic and electrochemical properties. Chem Commun 2014; 50: 15952–5. 10.1039/C4CC06064D 25381812

