# Supplementary material for: General synthesis of 2D rare-earth oxide single crystals with tailorable facets
Source: Natl Sci Rev. 2021 Aug 23;9(5):nwab153. doi: 10.1093/nsr/nwab153 (PMC9113103; doi:10.1093/nsr/nwab153)
Supplement: nwab153_Supplemental_File [file nwab153_supplemental_file.docx]

**Supplementary Information**

**General synthesis of 2D rare-earth oxides single crystals with tailorable facet**

*Linyang Li^1,†^, Fangyun Lu^1,†^, Wenqi Xiong^3^, Yu Ding^1^, Yangyi Lu^1^, Yao Xiao^2^, Xin Tong^1^, Yao Wang^1^, Shuangfeng Jia^3^, Jianbo Wang^3^, Rafael G. Mendes^4,5^, Mark H. Rümmeli^4,5,6,7^, Shengjun Yuan^3^, Mengqi Zeng^1,^* and Lei Fu^1,2,^**

^1^College of Chemistry and Molecular Sciences, Wuhan University, Wuhan 430072, China

^2^The Institute for Advanced Studies, Wuhan University, Wuhan 430072, China

^3^Key Laboratory of Artificial Micro- and Nano-structures of Ministry of Education and School of Physics and Technology, Wuhan University, Wuhan 430072, China

^4^College of Physics, Optoelectronics and Energy & Collaborative Innovation Center of Suzhou Nano Science and Technology, Soochow University, Suzhou 215006, China

^5^Institute for Complex Materials, IFW Dresden, Dresden 01069, Germany

^6^Centre of Polymer and Carbon Materials, Polish Academy of Sciences, Zabrze 41-819, Poland

^7^Institute of Environmental Technology, VSB-Technical University of Ostrava, Ostrava, 708 33, Czech Republic

***Corresponding author.** E-mail: zengmq_lan@whu.edu.cn; leifu@whu.edu.cn.

^†^Equally contributed to this work.

**METHODS**

**Transfer of 2D REO single crystals**

To transfer 2D REO onto various substrates such as SiO_2_/Si and TEM grids, the dry-transfer approach was adopted. Firstly, a 9 wt% polyvinyl alcohol (PVA) (Alfa Aesar, 98−99% hydrolyzed, high molecular weight) aqueous solution was spin-coated on a smooth substrate and then was dried in a vacuum at 60 ^o^C for 24 h. After that, the PVA film as a polymer mediator was obtained. Next, the PVA film was covered on the sample and heated to 100°C for 1 h. Then, the PVA film was split off from the growth substrate and transferred onto the target substrate. Finally, the polymer mediator was removed by dissolving in deionized water at room temperature overnight, and the 2D REO samples were transferred to the target substrate.

**DFT calculations**

DFT calculations were carried out using the Vienna Ab Initio Simulation Package (VASP) [[1](#_ENREF_1)]. The ion-electron interactions and exchange correlation potential were described from projected augmented wave (PAW) and generalized gradient approximation (GGA) [[2](#_ENREF_2)]. The kinetic energy cutoff and *k*-point meshes were set to 500 eV and 3 × 3 × 1, respectively [[3](#_ENREF_3)]. A vacuum thickness of 20 Å was added to avoid periodic interaction. The van der Waals interactions between Cl atoms and CeO_2_ crystal were treated by the semi-empirical DFT-D2 method [[4](#_ENREF_4),[5](#_ENREF_5)]. The onsite Coulomb correlation of Ce *4f* electrons was adopted within GGA+U (Hubbard parameter U=5.5 eV) [[6](#_ENREF_6)]. In all calculations, the atomic positions were completely optimized. The lattice constant of bulk CeO_2_ was 5.41 Å, in agreement with other reports [[7](#_ENREF_7)]. The surface unit cells were created by cutting bulk CeO_2_ along (111) and (100) orientations. Then, the Cl atoms were adsorbed onto supercell 3 × 3 (111) and 2 × 2 (100) with various coverages, as shown in Figure S24–26. The number of absorbed Cl atoms and the corresponding surface coverage for the CeO_2_ (111) and CeO_2_ (100) surfaces were described in Tables S2 and 3. The surface energy was calculated based on the equation: $\gamma=\left( E_{slab+Cl, relax}-\frac{N_{slab}}{N_{bulk}}E_{bulk}-N_{Cl}\mu_{Cl} \right)/A-\gamma_{frozen}$, where *E_slab+Cl, relax_* is the total energy of a relaxed slab with adsorbed Cl atoms, *N_slab_* is the number of atoms in the slab calculation, *N_bulk_* is the number of atoms in the bulk crystal calculation, *E_bulk_* is the energy of the bulk unit cell containing the same number of atoms as in the slab, A is the surface area, *γ_frozen_* is the surface energy of a surface with atom positions frozen to bulk values, and *N_Cl_* is the number of adsorbed Cl atoms.

**
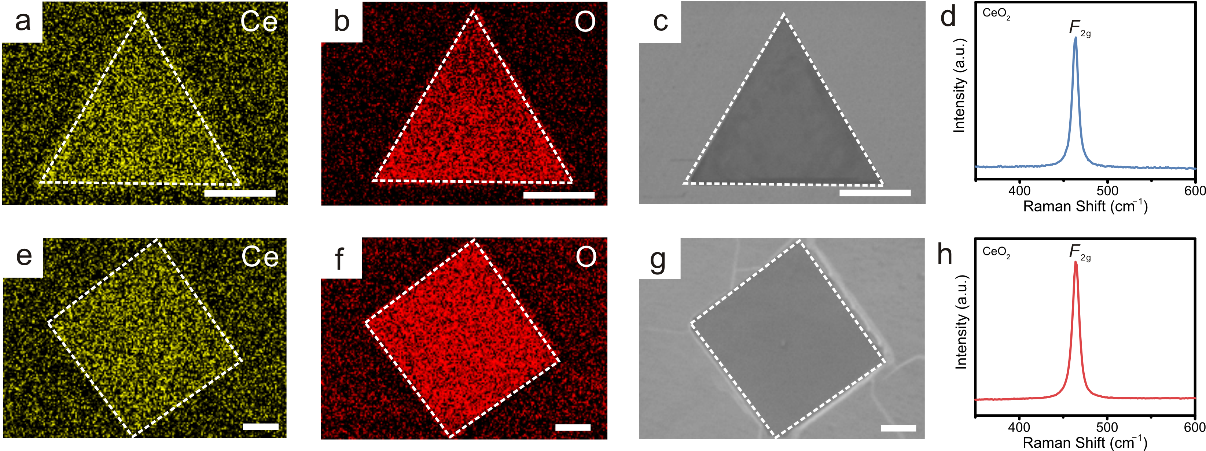
**

**Figure S1.** SEM EDS elemental mappings and Raman spectra of 2D CeO_2_ single crystals exposing different facets, scale bar: 1 μm.

Raman spectrum was employed to verify the structure of 2D CeO_2_ single crystals with different facets we obtained and scanning electron microscope (SEM) and energy-dispersive X-ray spectroscopy (EDS) mapping were also presented to confirm the crystal uniformity (Figure S1). In Supplementary Figure S1a–c and e–g, EDS elemental mappings of Ce and O were used to confirm the uniformity of the 2D CeO_2_ single crystals exposing (111) facet and (100) facet, which show regular triangle or square shape and uniform elemental distribution. The Raman spectra of a triangular and square crystal showed a single peak at ∼463 cm^−1^ and ~465 cm^−1^ (Figure S1d and 1h), which can be attributed to the *F*_2g_ mode of cubic phase CeO_2_, and the symmetry and strength of *F*_2g_ confirm the high crystallinity of the 2D CeO_2_ single crystals [[8](#_ENREF_8)].


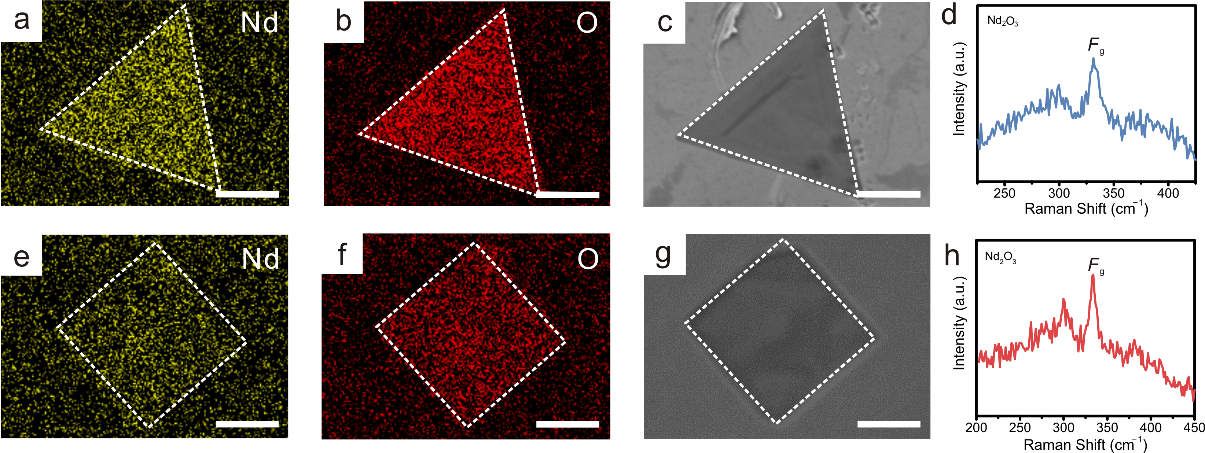


**Figure S2.** SEM EDS elemental mapping and Raman spectrum of 2D Nd_2_O_3_ exposing different crystal facet, scale bar: 1 μm.

In Figure S2a–c and e–g, EDS elemental mappings of Nd and O were used to confirm the uniformity of the 2D Nd_2_O_3_ single crystals exposing (111) facet and (100) facet, which showed regular triangle or square shape and uniform elemental distribution. The Raman spectra of a triangular and square crystal showed a single peak at ∼332 cm^−1^ and ~334 cm^−1^, respectively (Figure S2d and 2h), which can be attributed to the *F*_g_ mode of cubic phase Nd_2_O_3_, and the symmetry and strength of *F*_g_ confirm the high crystallinity of the 2D Nd_2_O_3_ single crystals [[9](#_ENREF_9)].


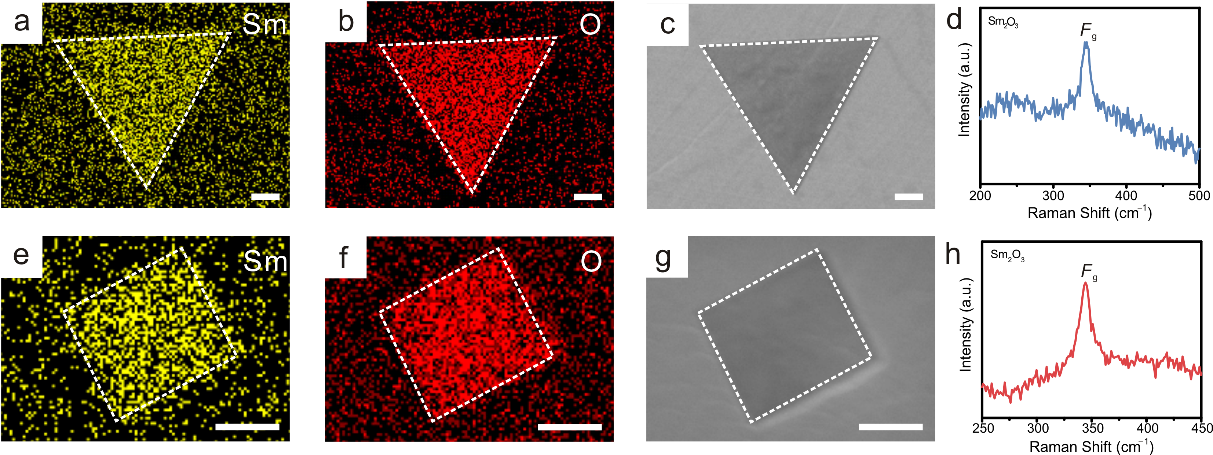


**Figure S3.** SEM EDS elemental mapping and Raman spectrum of 2D Sm_2_O_3_ exposing different crystal facets, scale bar: 0.5 μm.

In Figure S3a–c and e–g, EDS elemental mappings of Sm and O were used to confirm the uniformity of the 2D Sm_2_O_3_ single crystals exposing (111) facet and (100) facet, which show regular triangle or square shape and uniform elemental distribution. The Raman spectra of a triangular and square crystal showed a single peak at ∼344 cm^−1^ and ~343 cm^−1^, respectively (Figure S3d and 3h), which can be attributed to the *F_g_* mode of cubic phase Sm_2_O_3_, and the symmetry and strength of *F_g_* confirm the high crystallinity of the 2D Sm_2_O_3_ single crystals [[9](#_ENREF_9)].


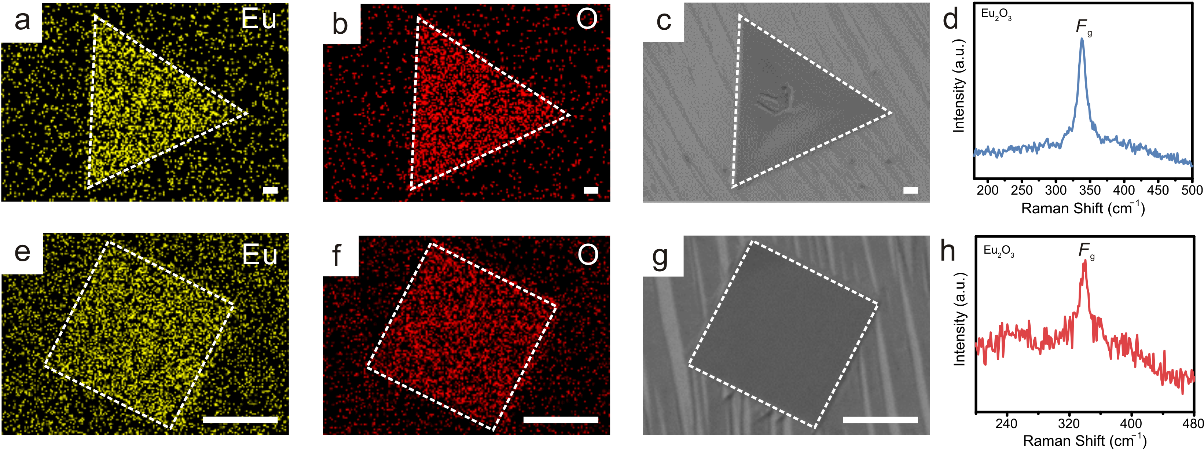


**Figure S4.** SEM EDS elemental mapping and Raman spectrum of 2D Eu_2_O_3_ exposing different crystal facets, scale bar: 1 μm.

As seen in Figure S4a–c and e–g, EDS elemental mappings of Eu and O were used to confirm the uniformity of the 2D Eu_2_O_3_ single crystals exposing (111) facet and (100) facet, exhibiting regular triangle or square shape and uniform elemental distribution. The Raman spectra of a triangular and square crystal showed a single peak at ∼338 cm^−1^ and ~339 cm^−1^, respectively (Figure S4d and 4h), which can be attributed to the *F_g_* mode of cubic phase Eu_2_O_3_, and the symmetry and strength of *F_g_* confirm the high crystallinity of the 2D Eu_2_O_3_ single crystals [[9](#_ENREF_9)].


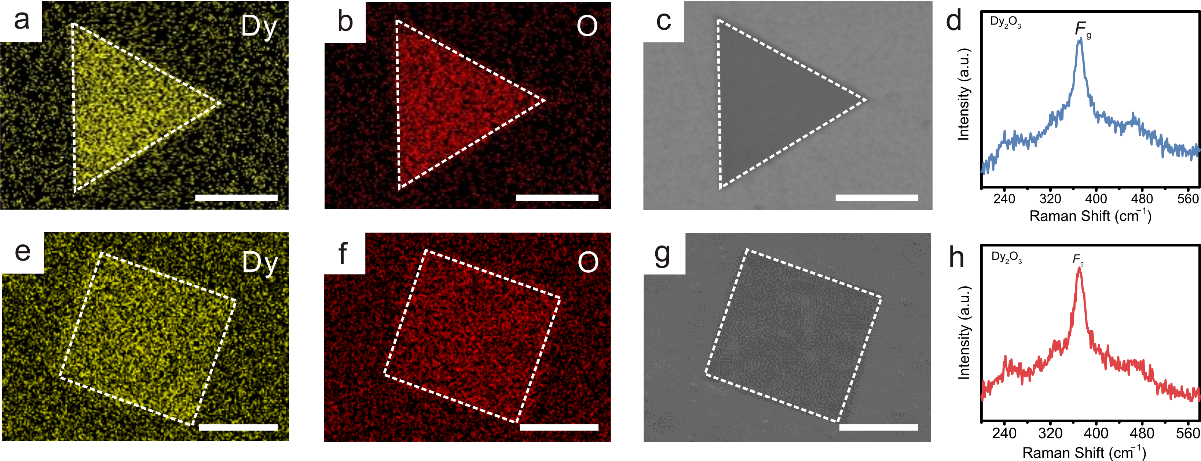


**Figure S5.** SEM EDS elemental mapping and Raman spectrum of 2D Dy_2_O_3_ exposing different crystal facets, scale bar: 0.5 μm.

EDS elemental mappings of Dy and O were used to confirm the uniformity of the 2D Dy_2_O_3_ single crystals exposing (111) facet and (100) facet, as seen in Figure S5a–c and e–g, both of which exhibit regular triangle or square shape and uniform elemental distribution. The Raman spectra of a triangular and square crystal showed a single peak at ∼370 cm^−1^ and ~371 cm^−1^ (Figure S5d and 5h), which can be attributed to the *F_g_* mode of cubic phase Dy_2_O_3_, and the symmetry and strength of *F_g_* confirm the high crystallinity of the 2D Dy_2_O_3_ single crystals [[9](#_ENREF_9)].

**
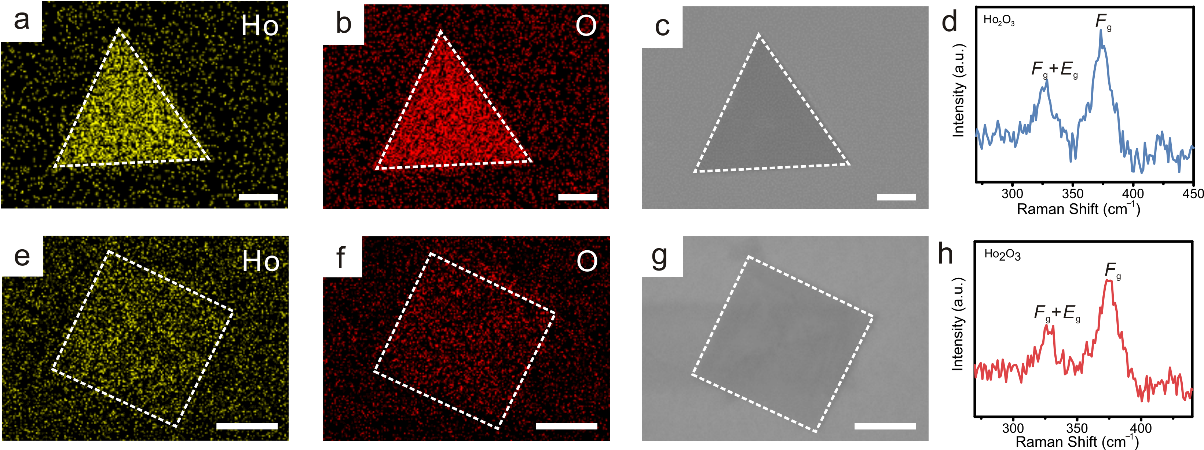
**

**Figure S6.** SEM EDS elemental mapping and Raman spectrum of 2D Ho_2_O_3_ exposing different crystal facets, scale bar: 0.5 μm.

EDS elemental mappings of Ho and O were used to confirm the uniformity of the 2D Ho_2_O_3_ single crystals exposing (111) facet and (100) facet, as seen in Figure S6a–c and e–g, both of which exhibit regular triangle or square shape and uniform elemental distribution. The Raman spectra of a triangular and square crystal showed peaks at ∼375 cm^−1^ (*F*_g_) and ~326 cm^−1^ (*F*_g_+*E*_g_) (Figure S6d and 6h), and the symmetry and strength confirm the high crystallinity of the 2D Ho_2_O_3_ single crystals [[9](#_ENREF_9),[10](#_ENREF_10)].

**
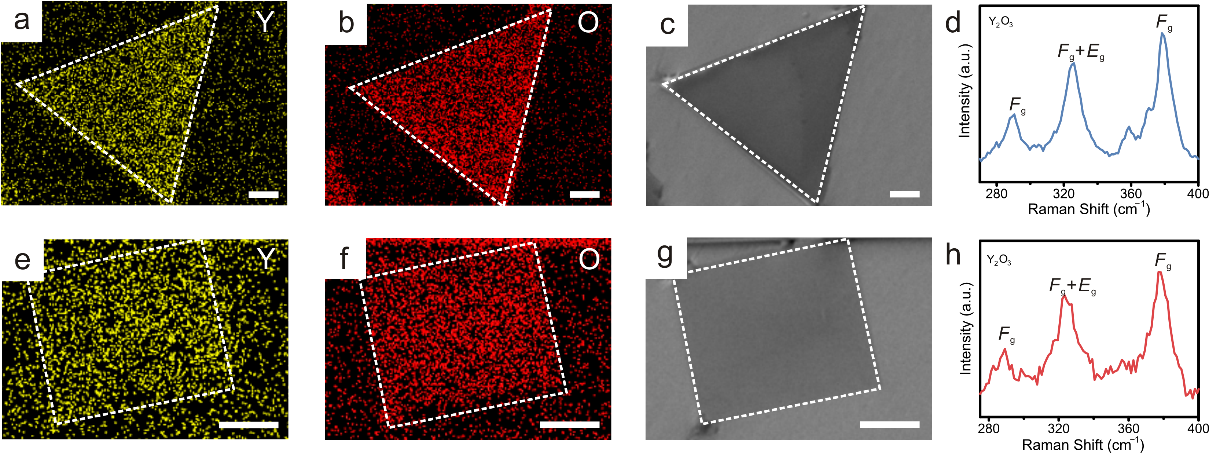
**

**Figure S7.** SEM EDS elemental mapping and Raman spectrum of 2D Y_2_O_3_ exposing different crystal facets, scale bar: 1 μm.

EDS elemental mappings of Y and O were used to confirm the uniformity of the 2D Y_2_O_3_ single crystals exposing (111) facet and (100) facet, as seen in Figure S7a–c and e–g, both of which exhibit regular triangle or square shape and uniform elemental distribution. The Raman spectra of a triangular and square crystal showed peaks at ∼290 cm^−1^, ∼325 cm^−1^, and ~379 cm^−1^ (Figure S7d and 7h), which can be attributed to the *F*_g_, *F*_g_+*E*_g_, and *F*_g_ modes of cubic phase Y_2_O_3_, and the symmetry and strength confirm the high crystallinity of the 2D Y_2_O_3_ single crystals [[9](#_ENREF_9),[10](#_ENREF_10)].


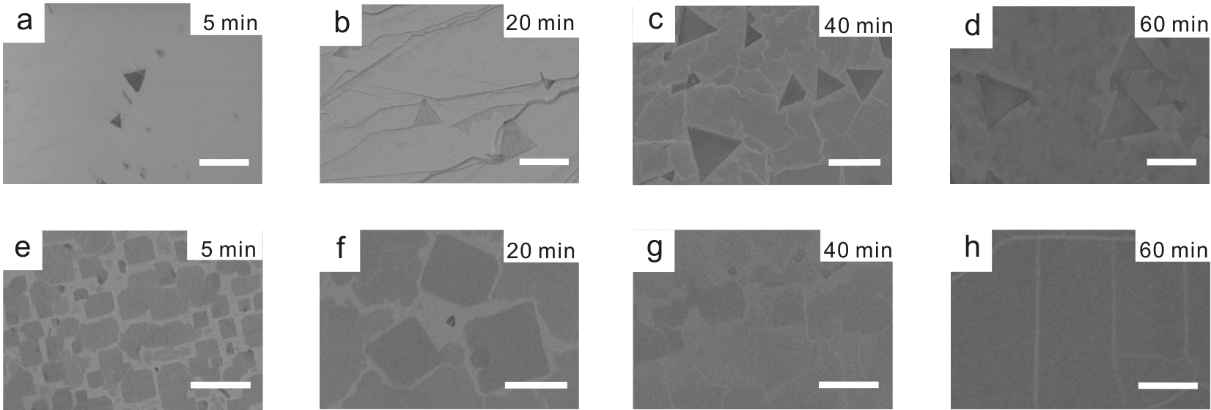


**Figure S8.** The growth of REO by controlling the reaction time. (a-d) SEM images of 2D CeO_2_(111) growth under different time. Scale bar: 2 μm. (e-f) SEM images of 2D CeO_2_(100) growth under different time. Scale bar: 5 μm.

The experiments of the growth of 2D CeO_2_ by controlling the reaction time ranging from 5 min to 60 min were conducted. As shown in Figure S8, the crystal size of both 2D CeO_2_(111) and 2D CeO_2_(100) becomes larger gradually by prolonging the reaction time. 2D CeO_2_(100) single crystals can even merge into a film. With the prolongation of time, more REO precipitates to interact with FCA, resulting in the enlargement of the lateral size because the growth of the non-layered material in 3D direction is effectively inhibited. In addition, we also found that the size of 2D CeO_2_(100) single crystals is larger than that of 2D CeO_2_(111). This comes the fact that during the growth of 2D CeO_2_(100) single crystals, more FCA is absorbed on the surface than that on 2D CeO_2_(111) single crystals, which will lead to a slower growth rate of CeO_2_(100) single crystals and is beneficial to obtain larger 2D CeO_2_(100) single crystals [[11](#_ENREF_11)].


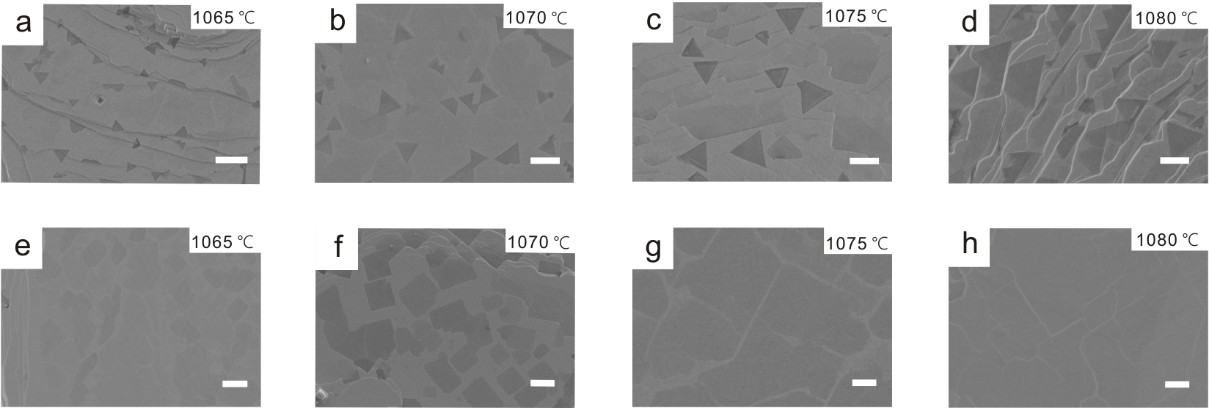


**Figure S9.** The growth of REO by controlling the reaction temperature. (a-d) SEM images of 2D CeO_2_(111) growth under different temperatures. (e-f) SEM images of 2D CeO_2_(100) growth under different temperatures. Scale bar: 2 μm.

The growth of 2D CeO_2_ at different temperatures ranging from 1065 ^o^C to 1080 ^o^C with the other parameters remaining the same were conducted. By regulating the reaction temperature, the crystal size of both 2D CeO_2_(111) and 2D CeO_2_(100) single crystals becomes larger gradually (Figure S9), which is consistent with the phenomenon reported in the literature [[12](#_ENREF_12)]. The higher temperature can lead to more effective nucleation and faster growth. During the growth of 2D CeO_2_(111) single crystals, due to the small amount of added FCA, its growth in the 3D direction cannot be sufficiently suppressed. Therefore, the crystal size becomes larger in the lateral direction while the thickness is not well controlled, which is not conducive to the enlargement of the lateral size. In terms of the growth of 2D CeO_2_(100) single crystals, more FCA can interact with the CeO_2_(100) facets, which effectively controls the thickness and is conducive to the expansion of the lateral size of the crystal and the subsequent crystal merging to form a film.

**
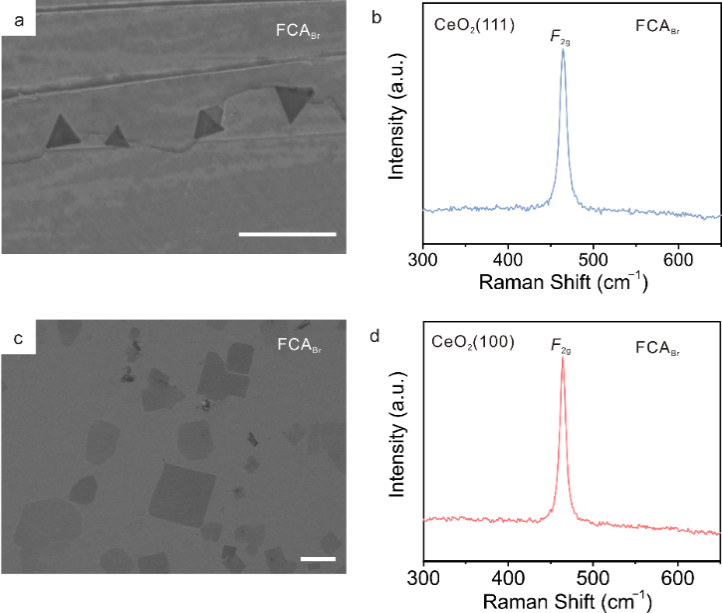
**

**Figure S10.** The universal facet controllable growth of 2D CeO_2_ single crystals by introducing FCA_Br_. (a, c) SEM images of 2D CeO_2_ single crystals exposing different facets by introducing FCA_Br_, respectively, scale bar: 3 μm. (b, d) Raman spectra of 2D CeO_2_ single crystals exposing (111) and (100) facet by introducing FCA_Br_, respectively.

**
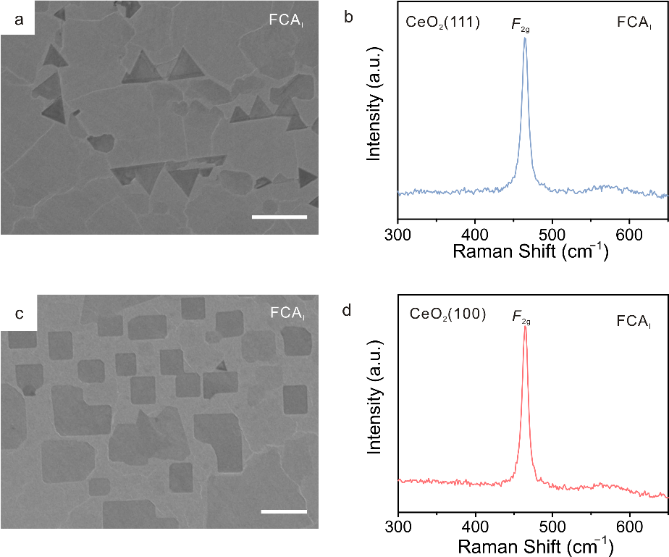
**

**Figure S11.** (a, c) SEM images of 2D CeO_2_ single crystals exposing different facets by introducing FCA_I_, respectively, scale bar: 3 μm. (b, d) Raman spectra of 2D CeO_2_ single crystals exposing (111) and (100) facet by introducing FCA_I_, respectively.

**
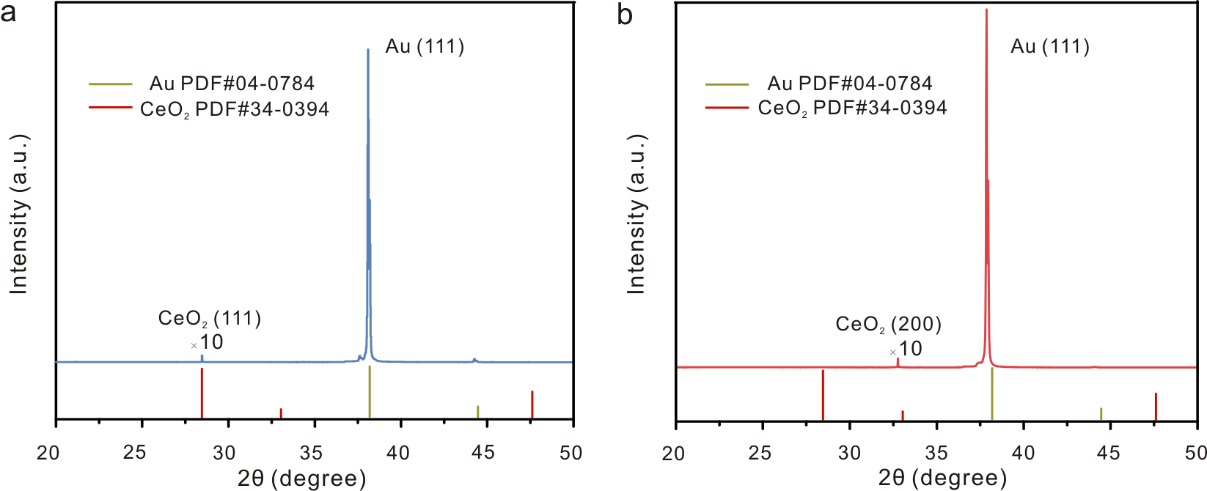
**

**Figure S12.** (a) XRD pattern of 2D CeO_2_ single crystals exposing (111) facet. (b) XRD pattern of 2D CeO_2_ single crystals exposing (100) facet.

As shown in Figure S12a, the only one detectable peak corresponding to (111) facet of cubic CeO_2_ in the X-ray diffraction (XRD) spectrum confirmed the successful synthesis of pure 2D CeO_2_ single crystals exposing (111) facet. In addition to Au substrate, no other signals attributed to the impurities were detected when the 2D CeO_2_(111) crystals are grown in a low *μ_Cl_* condition. A similar result was also observed for 2D CeO_2_(100) single crystals.

**
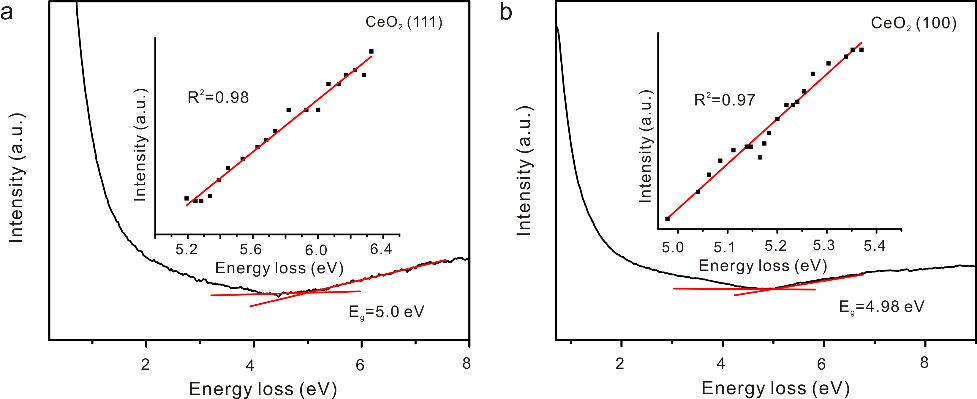
**

**Figure S13.** EELS of the 2D CeO_2_ single crystals exposing different facets. (a) EELS of 2D CeO_2_ single crystals exposing (111) facet. (b) EELS of 2D CeO_2_ single crystals exposing (100) facet.

Based on the low-loss electron energy loss spectrum (EELS) (Figure S13), the bandgap of the 2D CeO_2_(111) and CeO_2_(100) were estimated to be 5.0 eV and 4.98 eV.


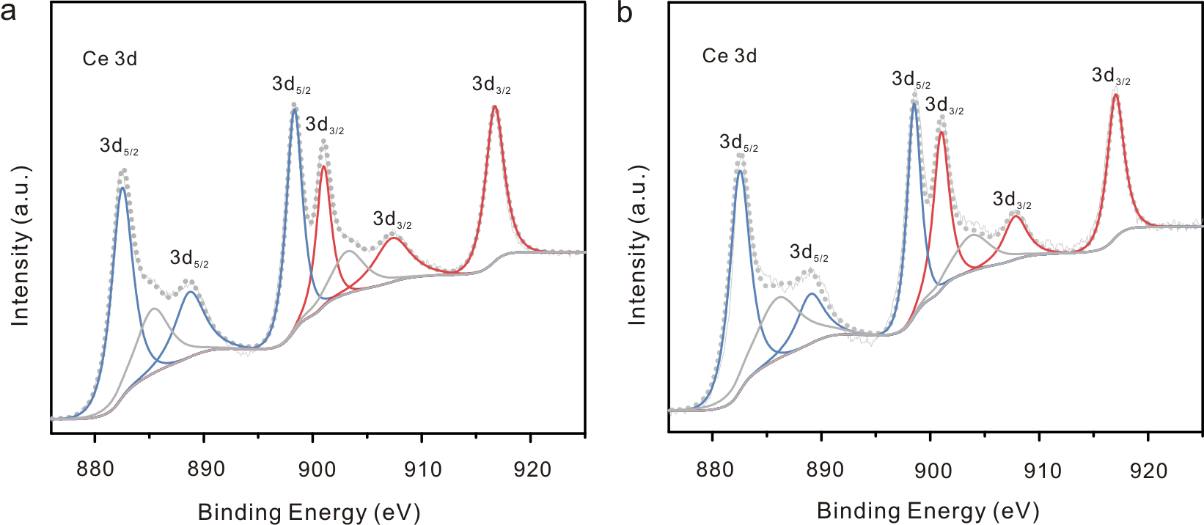


**Figure S14.** XPS analysis of Ce 3d orbitals and Cl 3d orbitals after the growth of REO by employing FCA_Cl_. (a) Ce 3d XPS of 2D CeO_2_ single crystals exposing (111) facet. (b) Ce 3d XPS of 2D CeO_2_ single crystals exposing (100) facet.

XPS spectra (Figure S14a–b) were collected to confirm the chemical composition of the CeO_2_ single crystals by employing NH_4_Cl as FAC. For 2D CeO_2_(111) single crystals (Figure S14a), peaks located at 882.5 eV and 917.0 eV correspond to the primary Ce 3d_5/2_ and Ce 3d_3/2_ states, while other doublets (at 889.0 eV, 898.0 eV; 901.0 eV, 907.8 eV) represent satellite features arising from the Ce 3d_5/2_ and Ce 3d_3/2_ ionization. The two peaks located at ~ 885 eV and ~ 904 eV are interpreted as being a manifestation of excited states of Ce^4+^. The chemical composition of CeO_2_ single crystals exposing (100) crystal facets was also determined (Figure S14b). The Ce 3d_5/2_ peak at 882.5 eV and 3d_3/2_ peak at 916.7 eV are in line with Ce^4+^. All these peaks are in accordance with the previous reports [[13](#_ENREF_13)].


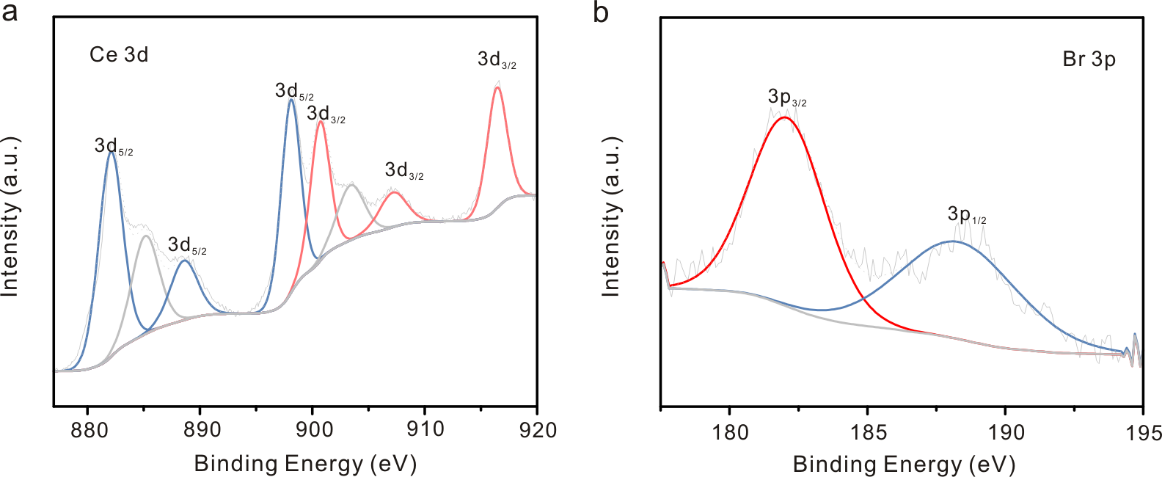


**Figure S15.** XPS analysis of Ce 3d orbitals and Br 3d orbitals after the growth of REO by employing FCA_Br_. (a) XPS analysis of Ce 3d orbitals after the growth of REO by employing FCA_Br_. (b) XPS analysis of Br 3d orbitals after the growth of REO by employing FCA_Br_.

XPS spectra (Figure S15a–b) were collected to confirm the chemical composition of the CeO_2_ single crystals by employing NH_4_Br as FAC. In terms of the introduction of FCA_Br_, peaks located at 882.1 eV and 916.4 eV correspond to the primary Ce 3d_5/2_ and Ce 3d_3/2_ states, while other doublets (at 888.6 eV, 898.1 eV; 900.7 eV, 907.2 eV) represent satellite features arising from the Ce 3d_5/2_ and Ce 3d_3/2_ ionization. The two peaks located at 885.1 eV and 903.4 eV are interpreted as being a manifestation of excited states of Ce^4+^. Meanwhile, the weak signal of Br on 2D CeO_2_(100) single crystals has been identified by XPS in Figure S15b, which demonstrates the existence of the facet controlling assistor (FCA_Br_). The two peaks located at 182.1 and 188.3 eV can be assigned to Br 3p_3/2_ and Br 3p_1/2_ states, which shift to a higher energy direction (~1 eV) [[14](#_ENREF_14)]. The Ce 3d peak shifted to the lower energy direction (~1 eV) after the adsorption of Br^−^ on the crystal surface, which confirmed the electron transfer from Br^−^ to Ce^4+^.


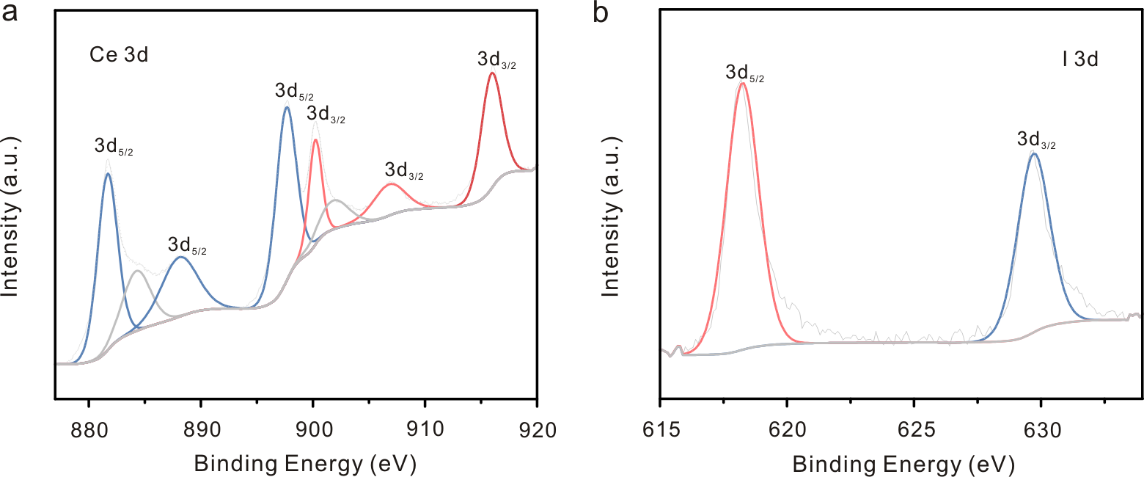


**Figure S16.** XPS analysis of Ce 3d orbitals and Br 3d orbitals after the growth of REO by employing FCA_I_. (a) XPS analysis of Ce 3d orbitals after the growth of REO by employing FCA_I_. (b) XPS analysis of Br 3d orbitals after the growth of REO by employing FCA_I_.

XPS spectra (Figure S16a–b) were collected to confirm the chemical composition of the CeO_2_ single crystals by employing NH_4_I as FAC. In terms of the introduction of FCA_I_, peaks located at 881.7 eV and 915.9 eV correspond to the primary Ce 3d_5/2_ and Ce 3d_3/2_ states, while other doublets (at 888.1 eV, 897.6 eV; 900.2 eV, 906.7 eV) represent satellite features arising from the Ce 3d_5/2_ and Ce 3d_3/2_ ionization. The two peaks located at 884.2 eV and 901.8 eV are interpreted as being a manifestation of excited states of Ce^4+^. Meanwhile, the weak signal of I on 2D CeO_2_(100) single crystals has been identified by XPS in Figure S16b, which demonstrates the existence of the facet controlling assistor (FCA_I_). The I 3d_5/2_ and 3d_3/2_ peaks are located at 618.3 and 629.7 eV [[15](#_ENREF_15)]. The Ce 3d peak shifted to the lower energy direction (~1 eV) after the adsorption of I^−^ on the crystal surface, which confirmed the electron transfer from I^−^ to Ce^4+^.


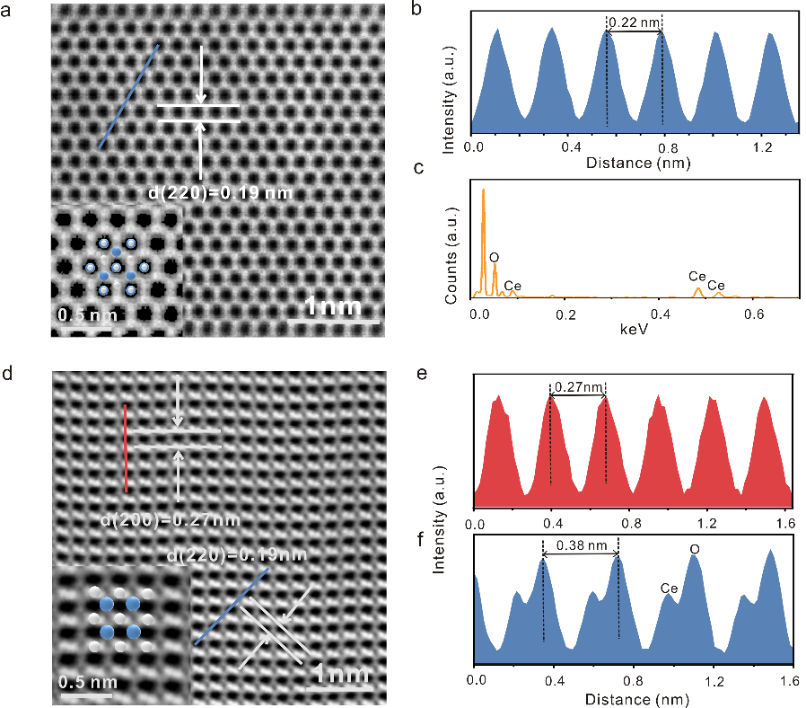


**Figure S17.** Atomic morphology of the as-synthesized 2D CeO_2_ single crystals. (a and d) BF–STEM images of 2D CeO_2_(111) and CeO_2_(100) single crystals, respectively (blue atoms: Ce; white atoms: O). (b) Intensity profile of the Ce and O atoms of 2D CeO_2_(111) collected from the region marked by a blue line in Figure S17(a). (c) EDS spectrum of the 2D CeO_2_(111) single crystal. (e) Intensity profile of the O atoms of 2D CeO_2_(100) collected from the region marked by a red line in Figure S17(d). (f) Intensity profile of the Ce, O atoms of 2D CeO_2_(100) collected from the region marked by a blue line in Figure S17(d).


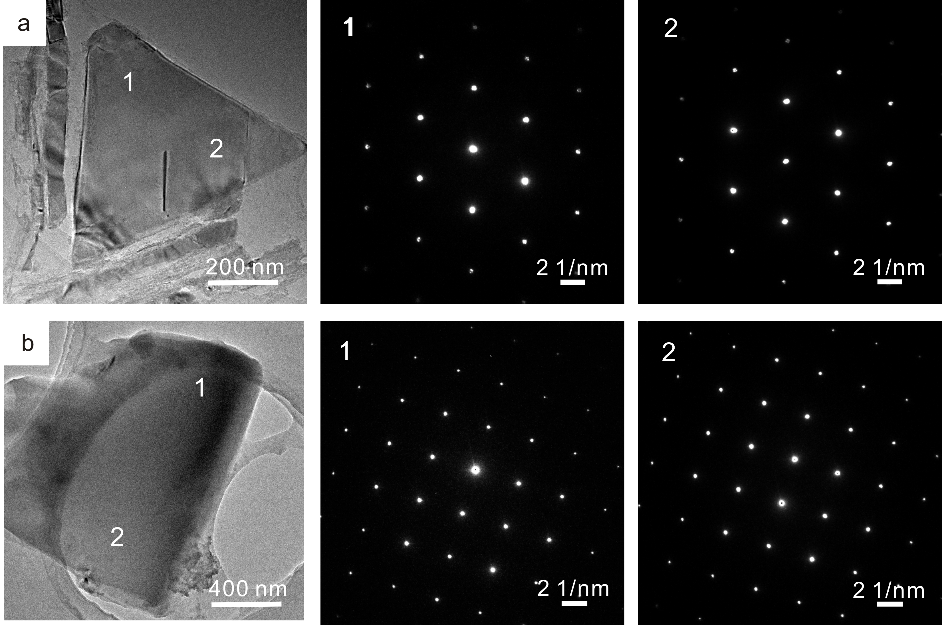


**Figure S18.** Demonstration of the single crystal property of 2D CeO_2_ crystals exposing different facets. (a, b) Low-magnification TEM images of 2D CeO_2_ single crystals exposing (111) and (100) facets, respectively. SAED patterns collected from the crystal shown in (a,b) at two different regions were shown in Figure S1, 2, correspondingly.

The single crystal property of 2D CeO_2_ crystal exposing (111) and (100) facets was identified by a series of selected area electron diffraction (SAED) patterns collected from different sites on the same CeO_2_ crystal, as seen in Figure S18. The sampling sites were marked in Figure S18a and b. All the diffractions in Figure S1 and S2 exhibits the same orientation, thus demonstrating that 2D CeO_2_ crystals were single crystals.


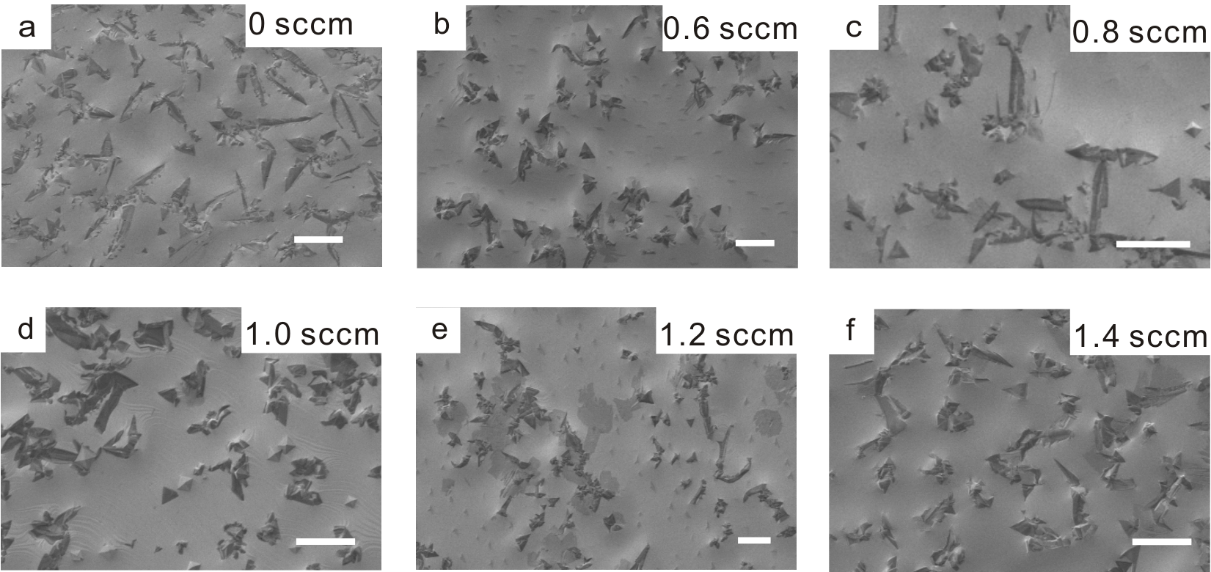


**Figure S19.** The control experiment of only introducing NH_3_ to the growth of REO. (a–f) SEM images of REO growth under different NH_3_ flow rates. Scale bar: 10 μm.

The control experiment of only introducing NH_3_ with different flow rates to the growth of REO was conducted (Figure S19). And it is verified that NH_3_ cannot act as FCA to realize the control of crystal facet in our growing system. When the amount of NH_3_ (at least 0.82 sccm) we introduced is comparable to that produced in the thermal decomposition of NH_4_Cl, the facet controllable growth of rare earth oxide single crystal is still unrealized. The reason may be contributed to that high temperature can cause NH_3_ gas to gradually decompose into various species, such as NH_2_, NH, N_2_, N, H_2_ and H [[16](#_ENREF_16),[17](#_ENREF_17)]. In our reaction temperature (1065 ^o^C–1080 ^o^C), NH_3_ will decompose to N_2_ and H_2_. N_2_ is considered and utilized as an inert gas and is a borderline base, which has a weak interaction with RE ions [[18](#_ENREF_18),[19](#_ENREF_19)]. Thus, it is difficult to realize the facet controlling process by introducing NH_3_. X^–^ derived from the thermal decomposition of NH_4_Cl is the main active agent for promoting the facet controllable growth of rare earth oxide single crystals.


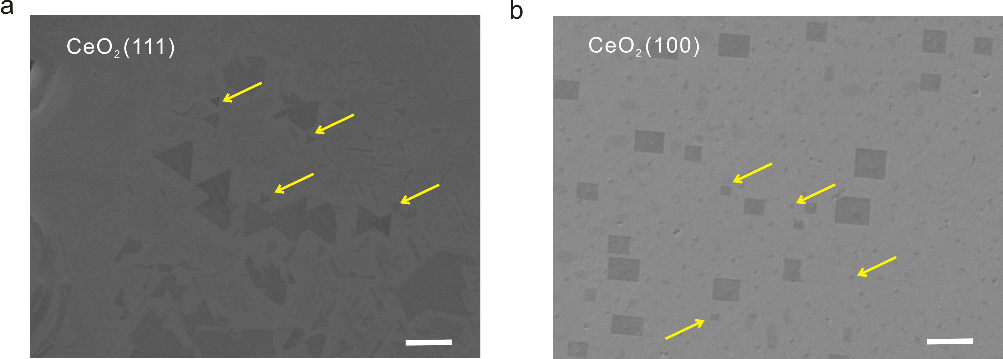


**Figure S20.** Demonstration of the early nucleation of 2D CeO_2_(111) and CeO_2_(100) single crystals (scale bar: 1μm).

The early nucleation of CeO_2_(111) and CeO_2_(100) single crystals were shown in Figure S20a and b, which exhibit small triangle and square morphology respectively marked by yellow arrows.


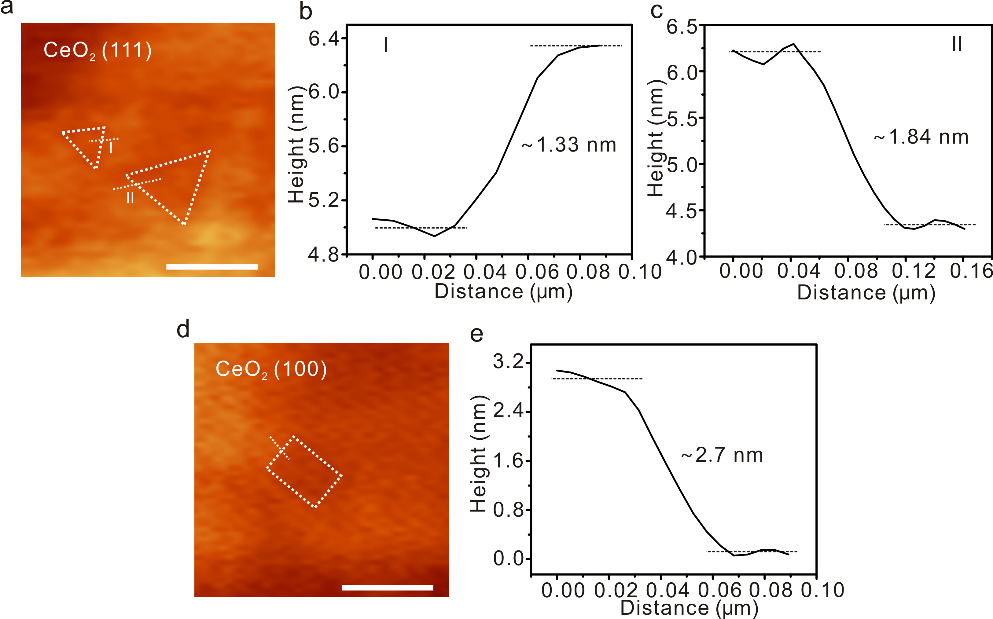


**Figure S21.** The thickness of the early nucleation of 2D CeO_2_(111) and CeO_2_(100) single crystals (scale bar: 0.3 μm).

The thickness of the early nucleation of CeO_2_(111) and CeO_2_(100) single crystals were shown in Figure S21, which reflects the realization of 2D nucleation of specific facets for the first time successfully.

**
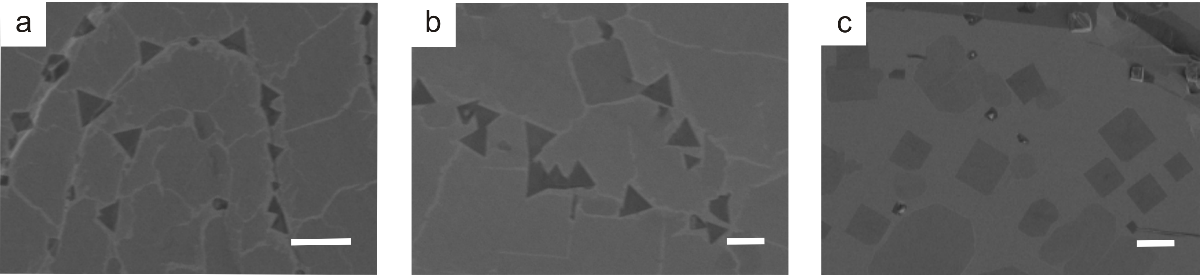
**

**Figure S22**. Evolution of the exposed facets of 2D CeO_2_ with the increasing concentration of FCA_Br_ (scale bar: 3 μm, a: *γ*_(111)_ < *γ*_(100)_; b: *γ*_(100)_ ≈ *γ*_(111)_; c: *γ*_(100)_ < *γ*_(111)_).

To explore the quantitative effect of different FCA on the process, we measured the weight of three different FCA (NH_4_X, X=Cl, Br, I) in the process. The weight and the corresponding shape of single crystals were recorded in Table S1. By increasing the concentration of different FCA, the shape of 2D CeO_2_ (111) and (100) single crystals changed from triangle to square (Figures 3d–f and S22–S23).


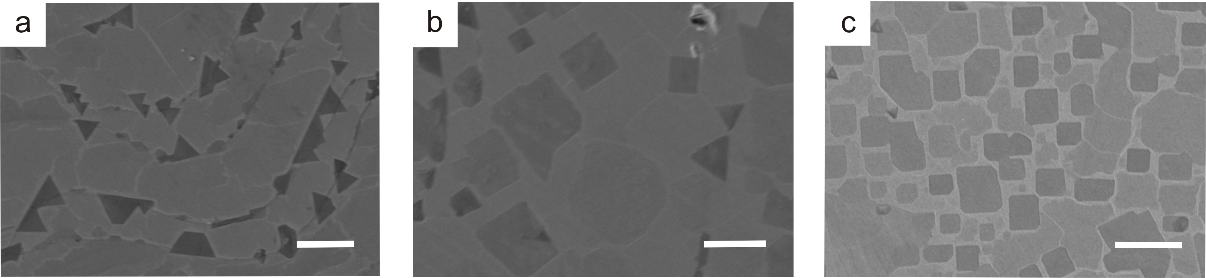


**Figure S23.** Evolution of the exposed facets of 2D CeO_2_ with the increasing concentration of FCA_I_ (scale bar: 3 μm, a: *γ*_(111)_ < *γ*_(100)_; b: *γ*_(100)_ ≈ *γ*_(111)_; c: *γ*_(100)_ < *γ*_(111)_).


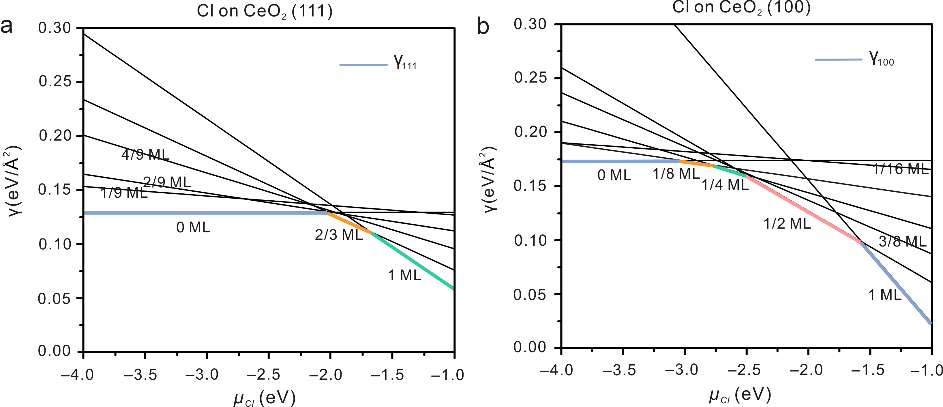


**Figure S24.** Surface energies of (a) CeO_2_(111) and (b) CeO_2_(100) with various Cl coverages as a function of chlorine chemical potential *μ_Cl_*, plotted as black lines with coverage labels. The bold colored lines indicate the surface coverage with the lowest surface energy at each chemical potential.

To investigate the mechanism of our strategy, DFT calculations were employed. The surface energy was calculated based on the equation of

$$\gamma=\left( E_{slab+Cl, relax}-\frac{N_{slab}}{N_{bulk}}E_{bulk}-N_{Cl}\mu_{Cl} \right)/A-\gamma_{frozen}$$

where E_slab+Cl, relax_ is the total energy of a relaxed slab with adsorbed Cl, N_slab_ is the number of atoms in the slab calculation, N_bulk_ is the number of atoms in the bulk crystal calculation, E_bulk_ is the energy of the bulk unit cell containing the same number of atoms as in the slab, A is the surface area, γ_frozen_ is the surface energy of a surface with atom positions frozen to bulk values, and N_Cl_ is the number of adsorbed Cl atoms.

We plot *γ* of CeO_2_(111) and CeO_2_(100) as a function of *μ_Cl_* (Figure S24). The optimized structures of CeO_2_(111) and CeO_2_(100) with various Cl surface coverages are shown in Figures S25 and S26. The number of absorbed Cl atoms and the corresponding surface coverage for the CeO_2_(111) and CeO_2_(100) surfaces were described in Tables S2 and S3, respectively.

**
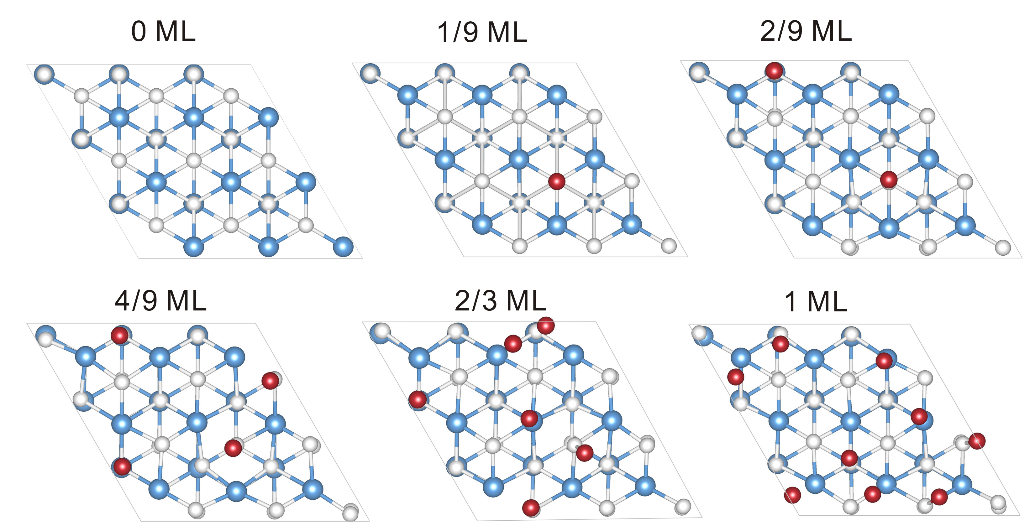
**

**Figure S25.** Optimized structures of CeO_2_ (111) with various Cl surface coverages. The blue, white, and red balls are Ce, O, and Cl atoms, respectively.

**
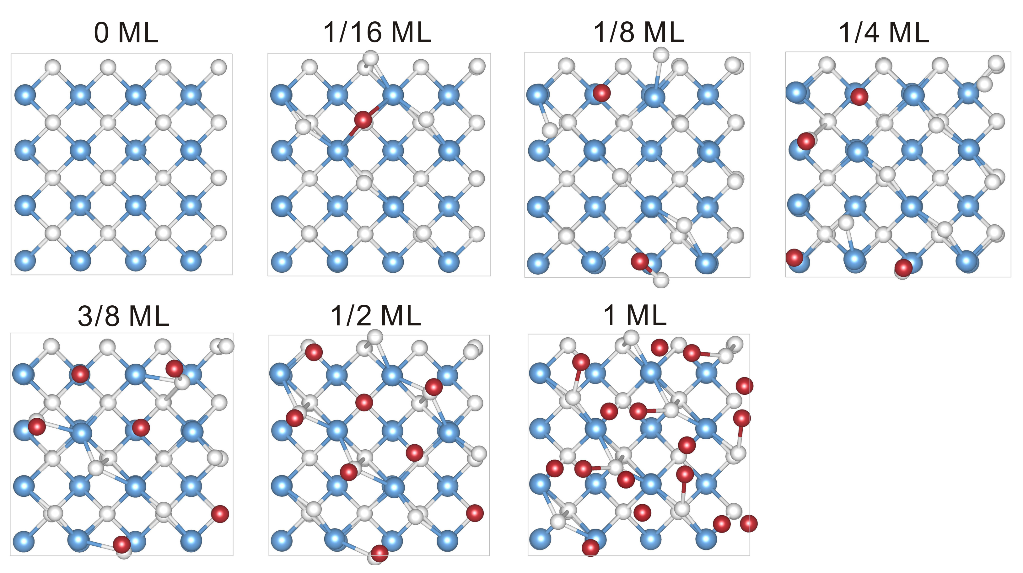
**

**Figure S26.** Optimized structures of CeO_2_ (100) with various Cl surface coverages. The blue, white, and red balls are Ce, O, and Cl atoms, respectively.


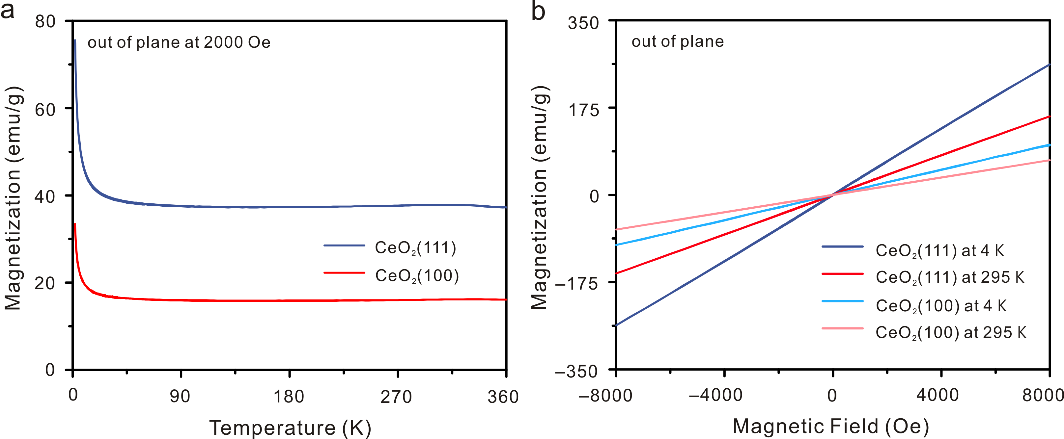


**Figure S27.** Magnetic characterization of CeO_2_ single crystals. (a) Out of plane temperature-dependent magnetization of CeO_2_(111) and CeO_2_(100) single crystals at 2000 Oe. (b) Out of plane magnetic hysteresis (M–H) loops of CeO_2_(111) and CeO_2_(100) single crystals at different temperatures.

The out of plane magnetic characterization of CeO_2_ single crystals. The variation trend of magnetic susceptibility with temperature conforms to Curie–Weiss law (Figure S27).

**
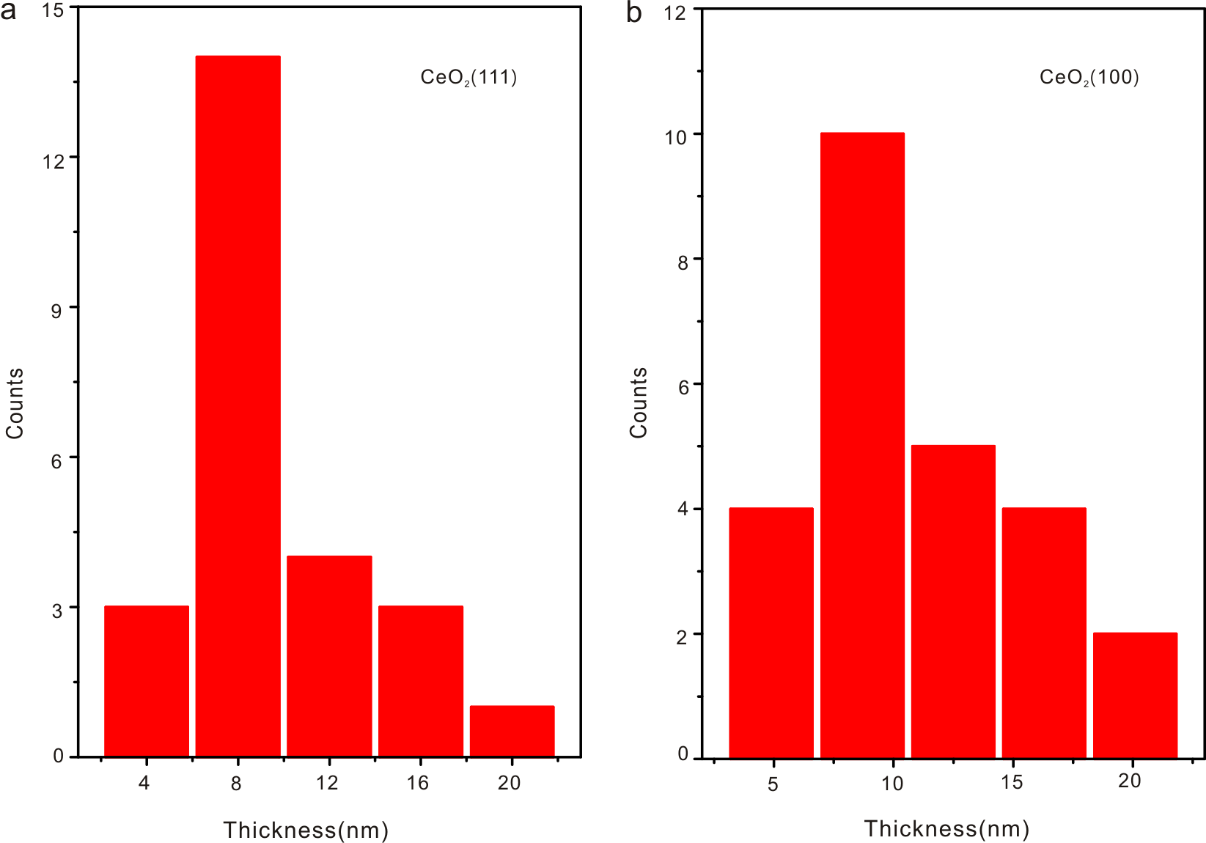
**

**Figure S28.** The statistical thickness data of 2D CeO_2_(111) single crystals (a) and CeO_2_(100) single crystals (b) on the substrate.

By employing the Five-point sampling method, we obtained the distribution of CeO_2_(111) single crystals and CeO_2_(100) single crystals in different areas on the substrate. Table S4 and S5 showed the areas of different areas and the coverage of CeO_2_(111) and CeO_2_(100) single crystals were calculated to be 30.16% and 52.33%, respectively. Then, we obtained the thickness of CeO_2_(111) and CeO_2_(100) single crystals on the substrate by AFM results. Figure S28a and b showed the average thickness of CeO_2_(111) and CeO_2_(100) single crystals were measured to be 9.02 nm and 10.70 nm, respectively. Thus, the weight of the CeO_2_(111) and CeO_2_(100) single crystals can be evaluated to be 6.98 × 10^‒7^ g and 1.34 × 10^‒6^ g.

**Table S1.** The weight of different FCA for the growth of CeO_2_(111) and CeO_2_(100) single crystals.

|  | m(FCA_Cl_)/g | m(FCA_Br_)/g | m(FCA_I_)/g |
| --- | --- | --- | --- |
| triangle | < 0.0010 | < 0.0024 | < 0.0150 |
| triangle & square | 0.0010 | 0.0024 | 0.0150 |
| square | 0.0015 | 0.0030 | 0.0200 |
| no crystals | 0.0030 | 0.0100 | 0.0300 |

**Table S2.** The number of absorbed Cl and their corresponding surface coverages for the CeO_2_ (111) surfaces.

| Number of Cl atoms | 0 | 1 | 2 | 4 | 6 | 9 |
| --- | --- | --- | --- | --- | --- | --- |
| Surface coverage of monolayer | 0 | 1/9 | 2/9 | 4/9 | 2/3 | 1 |

**Table S3.** The number of absorbed Cl and their corresponding surface coverages for the CeO_2_ (100) surfaces.

| Number of Cl atoms | 0 | 1 | 2 | 4 | 6 | 8 | 16 |
| --- | --- | --- | --- | --- | --- | --- | --- |
| Surface coverage of monolayer | 0 | 1/16 | 1/8 | 1/4 | 3/8 | 1/2 | 1 |

**Table S4.** The statistical data of CeO_2_(111) sample coverages on the substrate by Five-point sampling method.

| CeO_2_(111) | Sample area/µm^2^ | Total area/µm^2^ |
| --- | --- | --- |
| Top left | 724.439 | 2737.396 |
| Top right | 888.261 | 2737.341 |
| Middle | 851.477 | 2720.652 |
| Bottom left | 788.957 | 2738.548 |
| Bottom right | 863.253 | 2731.408 |

**Table S5.** The statistical data of CeO_2_(100) sample coverages on the substrate by the Five-point sampling method.

| CeO_2_(100) | Sample area/µm^2^ | Total area/µm^2^ |
| --- | --- | --- |
| Top left | 1134.555 | 2737.377 |
| Top right | 1929.231 | 2721.806 |
| Middle | 1176.510 | 2730.930 |
| Bottom left | 1421.617 | 2730.713 |
| Bottom right | 1482.174 | 2715.348 |

**REFERENCES**

1. Kresse G and Furthmuller J. Efficient iterative schemes for ab initio total-energy calculations using a plane-wave basis set. *Phys Rev B* 1996; **54**: 11169–86.

2. Perdew, JP, Burke, K and Ernzerhof, M. Generalized gradient approximation made simple. *Phys Rev Lett* 1996; **77**: 3865–8.

3. Monkhorst HJ and Pack JD. Special points for Brillouin-zone integrations. *Phys Rev B* 1976; **13**: 5188–92.

4. Grimme, S. Semiempirical GGA-type density functional constructed with a long-range dispersion correction. *J Comput Chem* 2006; **27**: 1787–99.

5. Kerber T, Sierka M and Sauer J. Application of semiempirical long-range dispersion corrections to periodic systems in density functional theory. *J Comput Chem* 2008; **29**: 2088–97.

6. Anisimov VI, Zaanen J and Andersen OK. Band theory and mott insulators: HubbardUinstead of StonerI. *Phys Rev B* 1991; **44**: 943–54.

7. Yang Z, Woo TK and Baudin M *et al.* Atomic and electronic structure of unreduced and reduced CeO_2_ surfaces: a first-principles study. *J Chem Phys* 2004; **120**: 7741–9.

8. Maensiri S, Masingboon C and Laokul P *et al.* Egg white synthesis and photoluminescence of platelike clusters of CeO_2_ nanoparticles. *Cryst Growth Des* 2007; **7**: 950–5.

9. Abrashev MV, Todorov ND and Geshev J. Raman spectra of R_2_O_3_ (R—rare earth) sesquioxides with C-type bixbyite crystal structure: A comparative study. *J Appl Phys* 2014; **116**: 103508.

10. Dilawar N, Mehrotra S and Varandani D *et al.* A Raman spectroscopic study of C-type rare earth sesquioxides. *Mater Charact* 2008; **59**: 462–7.

11. Ghosh S and Manna L. The many “facets” of halide ions in the chemistry of colloidal inorganic nanocrystals. *Chem Rev* 2018; **118**: 7804–64.

12. Cai Z, Liu B and Zou X *et al.* Chemical vapor deposition growth and applications of two-dimensional materials and their heterostructures. *Chem Rev* 2018; **118**: 6091–133.

13. Burroughs P, Hamnett A and Orchard AF*, et al.* Satellite structure in the X-ray photoelectron spectra of some binary and mixed oxides of lanthanum and cerium. *J Chem Sot., Dalton Trans* 1976: 1686–98.

14. Thibaut E, Boutique JP and Verbist JJ*, et al.* Electronic structure of uranium halides and oxyhalides in the solid state. An x-ray photoelectron spectral study of bonding ionicity. *J Am Chem Soc* 1982; **104**: 5266–73.

15. Morgan, WE, Van Wazer, JR and Stec, WJ. Inner-orbital photoelectron spectroscopy of the alkali metal halides, perchlorates, phosphates, and pyrophosphates. *J Am Chem Soc* 1973; **95**: 751-5.

16. Nabi G, Cao C and Khan WS *et al.* Synthesis, characterization, growth mechanism, photoluminescence and field emission properties of novel dandelion-like gallium nitride. *Appl Surf Sci* 2011; **257**: 10289–93.

17. Quah HJ, Hassan Z and Yam FK *et al.* Effects of ammonia-ambient annealing on physical and electrical characteristics of rare earth CeO_2_ as passivation film on silicon. *J Alloys Compd* 2017; **695**: 3104–15.

18. Achiwawanich S, James BD and Liesegang J. XPS and ToF-SIMS analysis of natural rubies and sapphires heated in an inert (N_2_) atmosphere. *Appl Surf Sci* 2007; **253**: 6883–91.

19. Koch, E-C. Acid-base interactions in energetic materials: I. The hard and soft acids and bases (HSAB) principle-insights to reactivity and sensitivity of energetic materials. *Propell Explos Pyrot* 2005; **30**: 5–16.
